# N^6^-methyladenosine and its epitranscriptomic effects on hematopoietic stem cell regulation and leukemogenesis

**DOI:** 10.1186/s10020-024-00965-x

**Published:** 2024-11-04

**Authors:** Kao-Jung Chang, Li-Yang Shiau, Shiuan-Chen Lin, Han-Ping Cheong, Ching-Yun Wang, Chun Ma, Yan-Wen Liang, Yi-Ping Yang, Po-Shen Ko, Chih-Hung Hsu, Shih-Hwa Chiou

**Affiliations:** 1 Department of Medical Research, Taipei Veterans General Hospital, Taipei, Taiwan; 2 Department of Ophthalmology, Taipei Veterans General Hospital, Taipei, Taiwan; 3 School of Medicine, National Yang Ming Chiao Tung University, Taipei, Taiwan; 4 Institute of Pharmacology, National Yang Ming Chiao Tung University, Taipei, Taiwan; 5 Department of Medical Education, Taichung Veterans General Hospital, Taipei, Taiwan; 6 Department of Life Sciences and Institute of Genomic Sciences, National Yang-Ming University, Taipei, Taiwan; 7 Division of Hematology, Department of Medicine, Taipei Veterans General Hospital, Taipei, Taiwan; 8 The Fourth Affiliated Hospital, and Department of Environmental Medicine, Women’s Hospital, Zhejiang University School of Medicine, Hangzhou, China; 9 Institute of Genetics, International School of Medicine, Zhejiang University, Hangzhou, China

**Keywords:** m6A, HSCs, Lymphocytes, Leukemia, Lymphoma

## Abstract

N6-methyladenosine (m6A) RNA modification orchestrates cellular epitranscriptome through tuning the homeostasis of transcript stability, translation efficiency, and the transcript affinity toward RNA-binding proteins (RBPs). An aberrant m6A deposition on RNA can lead toward oncogenic expression profile (mRNA), impaired mitochondrial metabolism (mtRNA), and translational suppression (rRNA) of tumor suppressor genes. In addition, non-coding RNAs (ncRNAs), such as X-inactive specific transcript (XIST), miRNAs, and α-ketoglutarate-centric metabolic transcripts are also regulated by the m6A epitranscriptome. Notably, recent studies had uncovered a myriad of m6A-modified transcripts the center of hematopoietic stem cell (HSC) regulation, in which m6A modification act as a context dependent switch to the on and off of hematopoietic stem cell (HSC) maintenance, lineage commitment and terminal differentiation. In this review, we sequentially unfold the m6A mediated epithelial-to-hematopoietic transition in progenitor blood cell production, lymphocytic lineage expansion (T cells, B cells, NK cells, and non-NK ILCs), and the m6A crosstalk with the onco-metabolic prospects of leukemogenesis. Together, an encompassing body of evidence highlighted the emerging m6A significance in the regulation of HSC biology and leukemogenesis.

## Introduction

Epigenetics by its literal definition, refers to the copious machinery that reshapes gene expression profiles without altering the genetic sequence. Among the manifold of epigenetic mechanisms, RNA modifications have emerged as a trending research prospect that parallels histone and DNA modifications as the key paradigm that dominates the cell fate decision process. Hematopoiesis in particular, is a complex biology process that actively recruits histone, DNA and RNA modification to coordinate hematologic cell expansion, differentiation and crosstalk. In this article, we review how N6-methyladenosine (m6A) participate in the regulation of hematopoiesis, the de-differentiation process of endothelial-to-hematogenic transition (EHT), the differentiation of HSCs to lymphoid lineage, and the accumulative cues that confers leukemogenic events under the m6A epitranscriptomic effects.

In a step-by-step manner, we first introduce the basic prospects of m6A physiology, including how it is biochemically installed, removed, and being bound by reader proteins. Moreover, we explored the up-to-date m6A detection methods that employs chemical or affinity-based enrichment sequencing methods. Then a list of m6A-related players was highlighted with their particular contribution to the HSC biology. Dysregulation of m6A can disrupt normal hematopoiesis and lead toward either cytopenic or leukemic conditions. While the leukemic events were often lethal and hard to treat, we in depth report how m6A effects confers the leukemogenic milieu of acute lymphoblastic leukemia, chronic lymphocytic leukemia, and lymphoma. Last but not least, we think it being interesting to point out that the m6A-mediated aberrant immune cell activation and the m6A crosstalk with *Xist* RNA may conjointly foster an immunity condition that favors autoimmune diseases such as systemic lupus erythematosus (SLE) and rheumatoid arthritis (RA). In sum, we have collected a robust body of real-world disease models, in which different aspects of evidence had confirmed that the m6A modification are implicated in various hematological physiologic and pathologic regulation.

## Basic m6A regulatory machinery

### Epigenetics and m6A modification

Epigenetic regulation had been previously linked toward HSC regulation through the well-established histone-DNA modification apertures. The identification of histone and DNA modification enzymes and epigenetic mark readers, had prompted pharmaceutical investments in targeting draggable epigenetic enzymes (Miranda Furtado et al. 2019), so as to treat leukemic diseases (Dawson et al. 2012).

Recent studies (An and Duan 2022; Uddin et al. 2021) had pointed out that the RNA modification, in apart from the histone and DNA modification, act as a molecular hinge to connect upstream signaling cues and the downstream cellular effects. Biochemically, the RNA modifications are much flexible than chromatin modifications in terms of exerting transient effects or undertaking vast and fast cellular programming (Batista et al. 2014; Commerford et al. 1982). And in the case of hematopoiesis, the nimble RNA modification switch shut down gene expression through targeted transcript degradation and translation quenching (Wang et al. 2018a; Mapperley et al. 2021), these post-transcriptional activity help blood lineages swiftly redirect transcriptome momentum, and integrates endogenous programming (HSC expansion, lineage commitment) and exogenous signals (immunity maturation, infectious stimuli) without rebooting the whole expression process starting from chromatin re-organization, polymerase assembly and transcription completion. Therefore, the RNA modification was an energy conservative mechanism that facilitates individual cells to cope with multifarious biological demands (Boo and Kim 2020; Jonkhout et al. 2017).

N6-methyladenosine (m6A) stands out as the single most prevalent RNA modification that covers a wide range of RNA species (mRNA, miRNA, snRNA, snoRNA, rRNA, and ncRNA) in the eukaryotic transcriptome (Shi et al. 2019; Liu and Pan 2016; Delaunay and Frye 2019). Structurally, m6A is an adenosine molecule with methylated nitrogen attached to the 6th carbon of the adenine moiety. Approximately 0.1–0.4% of total adenosine molecules are modified by m6A, accounting for 50% of all methylated ribonucleotides, regardless of the type (Wei et al. 1975). m6A is typically deposited at RRA*CH motifs (R = A or G; H = A, C, or U), with the asterisk marking the primary m6A site. MeRIP-seq, a high throughput sequencing of the m6A signals, has shown that m6A signals are enriched in mRNA regions such as long exons, stop codon periphery, and 3' UTRs. The uneven distribution of m6A nucleotides across the transcriptome landscape reflects its role in modulating region-specific functions like RNA splicing, cap-independent translation, mRNA deadenylation, endoribonucleolytic cleavage, and transcript translocation. These m6A events collectively orchestrate gene expression and modulate cell homeostasis in self-renewal, clonal expansion, and lineage commitment.

### Methods to study m6A modifications

**Fig. 1:**
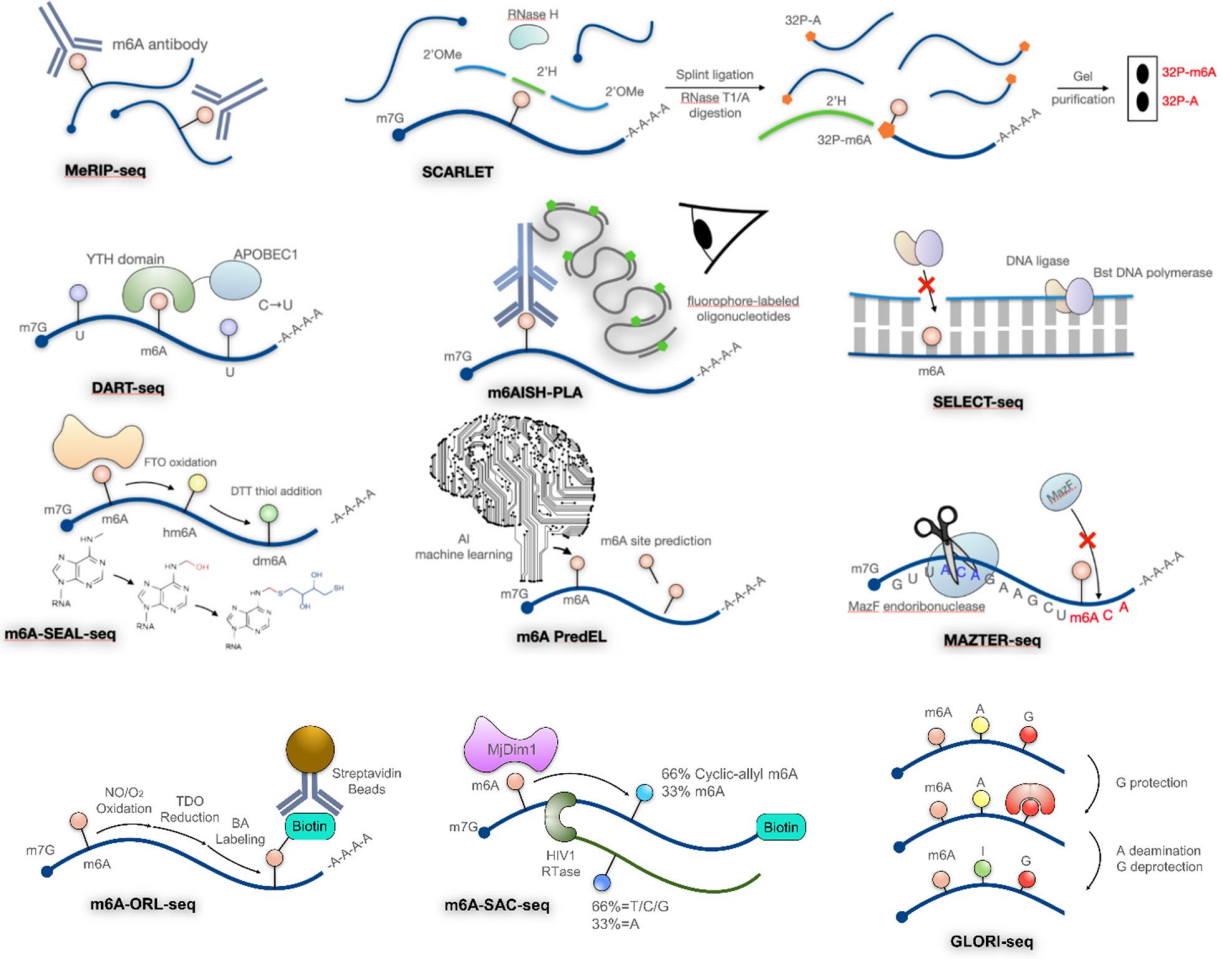
Summary of high-throughput methods to study m6A modifications

To explore how m6A landscapes modify gene expression and affect cell behavior, multiple techniques have emerged to locate m6A sites in the transcriptome. These techniques can be categorized into three main approaches: antibody-dependent immunoprecipitation, specific enzyme binding, and chemical-based detection (Fig. 1, Table 1). Methylated RNA immunoprecipitation sequencing (MeRIP-seq) was the first developed and the most widely-used approach (Dominissini et al. 2012). However, it only detects m6A at a resolution of 100–200 nucleotides. Therefore, other immunoprecipitation methods such as photo-crosslinking-assisted m6A sequencing (PA-m6A-seq) (Chen et al. 2015), cross-linking immunoprecipitation sequencing (CLIP-seq) (Kuksa et al. 2017), m6A individual-nucleotide resolution cross-linking immunoprecipitation sequencing (miCLIP-seq) (Grozhik et al. 2017), and m6A level and isoform-characterization sequencing (m6A-LAIC-seq) (Molinie et al. 2016) improved resolution. Moreover, m^6^A-specific in situ hybridization mediated proximity ligation assay (m6AISH-PLA), which utilizes m6A antibodies, can acquire single-cell resolution imaging of m6A-modified RNA (Wang et al. 2022). However, these methods may have poor reproducibility due to the nonspecific binding of m6A antibodies. To address this, antibody-independent methods have been developed. Specific enzymes are applied in methods such as MAZTER-seq (Garcia-Campos et al. 2019), diversity arrays technology sequencing (DART-seq) (Meyer 2019), and m6A-SEAL-seq (Wang et al. 2020a) to identify m6A distribution. MAZTER-seq utilizes bacterial MazF endoribonuclease to cleave RNA before “ACA” sequence but not at “m6ACA” sequences; m6A-SEAL-seq employs FTO-assisted selective chemical labeling by dithiothreitol (DTT)-mediated thiol-addition; also, DART-seq uses YTH-APOBEC1 fusion protein to locate modified adenosines and change adjacent cytosines into uracils. Nevertheless, these methods often depend on transfection efficiency or are limited to low resolution. Chemical-based detection methods such as SCARLET identify m6A locations with single nucleotide resolution in mRNAs or lncRNAs (Liu et al. 2013); SELECT exploits the ability of m6A modification in RNA to hinder the elongation of DNA polymerases and nick ligation activity of ligases (Xiao et al. 2018); m6A-label-seq substitutes m6A with N6-allyladenosine (a6A) using allyl-SeAM and allyl-SAM before NGS to detect transcriptome-wide m6A sites (Shu et al. 2020). The m6A-ORL-seq is based on biotin labeling of m6A followed by strepavidin pull-down (Xie et al. 2022). The latest chemical-based detection, GLORI-seq, imitates the principle and workflow of bisulfite sequence (Liu et al. 2023). GLORI-seq converts unmethylated adenosine residue into inosine. Thus, identified adenosine residues in sequencing are residues with modification. Nevertheless, this approach cannot distinguish m6A from other adenosine methylation types, such as m6Am or m1A. Researchers must further process the data to remove such interference based on the features of m6Am or m1A. Concurrently, computational models such as M6APred-EL (Wei et al. 2018) and m6Anet (Hendra 2022) have been developed for m6A site prediction, identification, and quantification. These high-throughput sequencing methods allow for better examination of the chemical properties of m6A.

**Table 1:** Summary of high-throughput methods to study m6A modifications

Technique	Materials	Solve what problem	Limitation	Refs.
MeRIP (2012)	m6A antibody	Identify transcriptional-wide m6A landscape	Cannot identify residue specific m6A, quantitative m6A analysis was ineligible (m6A-LAIC-seq)	Dominissini et al. (2012)
SCARLET (2013)	Radioactive-labeling	Determines m6A status at single nucleotide solution in mRNA/lncRNA	Time-consuming and require radiolabeling	Liu et al. (2013)
PA-m6A-seq (2015)	m6A antibody	More accurately define sites with m6A modification	Antibody required	Chen et al. (2015)
m6A-LAIC-seq (2016)	m6A antibody	Allowing quantification of m6A levels (the ratio of methylated to non-methylated transcripts) on different isoforms	Antibody required	Molinie et al. (2016)
miCLIP (2017)	m6A antibody	identify specific m6A residues	antibody required	Grozhik et al. (2017)
SELECT (2018)	dTTP or dNTP, Bst DNA polymerase, ligase	No antibody required, single nucleotide resolution		Xiao et al. (2018)
m6APred-EL (2018)	Machine learning	A novel machine learning-based predictor predicts exact m6A sites		Wei et al. (2018)
m6A-CLIP (2019)	m6A antibody	Single nucleotide resolution, low input RNA required	Antibody required	Hsu and He (2019)
m6A–REF-seq (2019)	m6A-sensitive RNA endoribonuclease MazF	No antibody required, single nucleotide resolution	Dependent on m6A sequence or cellular transfection	Zhang et al. (2019)
MAZTER-seq (2019)	Bacterial single-stranded ribonuclease MazF	No antibody required, single nucleotide resolution	Dependent on m6A sequence or cellular transfection RNA secondary structure can greatly affect accuracy	Garcia-Campos et al. (2019)
DART-seq (2019)	YTH-APOBEC1 fusion protein	Low imput RNA required, reduced cross reactivity to other RNA modifications	Dependent on m6A sequence or cellular transfection	Meyer (2019)
m6A-SEAL-seq (2020)	FTO-assisted selective chemical labeling	Selectively label the m6A oxidation production, hm6 A, for m6 A detection, antibody free	Not single base resolution	Wang et al. (2020a)
m6AISH-PLA (2021)	m6A antibody, molecular beacon (MB)-based PLA	Cellular imaging of m6A RNA, allowing to identify m6A modification at specific location in RNAs and image m6A RNA with single-cell and single-molecule resolution		Ren et al. (2021)
m6A-ORL-Seq (2022)	Biotin labeling of m6A followed by pulldown through Streptavidin-conjugated beads	Applicability on sample with low methylation level. Higher reproducibility	Higher mutation rate (A to G) in bioinformatic analysis	Xie et al. (2022)
m6A-SAC-seq (2022)	Enzyme (MjDim1) treatment and iodine cyclization of m6A to create identifiable mismatched site in reverse transcription	Quantitative method	Efficacy depends on the motif surrounding m6A site	Ge et al. (2023)
GLORI-seq (2022)	Chemical conversion of unmethylated adenosine to inosine	Possibility to achieve absolute m6A quantification. Quantitative method at single base resolution	Unable to directly distinguish m6A from other adenosine modifications such as m6Am or m1A. Requires extra data processing steps to remove such interference	Liu et al. (2023; Shen et al. (2024)

The m6A RNA regulatory machinery is organized similarly to the molecular machinery responsible for other epigenetic mark, such as histone and DNA modifications. Namely, it includes the writers responsible for depositing of m6A marks (methyltransferases), readers that recognize m6A marks and modulate their functional effects, and erasers that remove the marks. Methyl-RNA immunoprecipitation sequencing (MeRIP-seq) also identified m6A modifications on apoptotic (*TNFR2*, *AXIN2*), stemness (*NOTCH1A*, Wnt target genes) and pro-differentiation (*HOXB4*, *MYB*, *MYC*, *BCL2*, *PTEN*) genes. To better understand how the m6A regulates blood development by targeting m6A-modified transcripts, we detailed the biochemical and stereochemical aspects of the m6A regulators.

### m6A writers

m6A deposition on mRNA is catalyzed by the methyltransferase complex (MTC), which consists of the catalytic subunit METTL3 and other accessory subunits, including METTL14, ZC3H13, WTAP, RBM15, VIRMA (also known as KIAA1429), and HAKAI. The assembly of MTC begins with the formation of the heterodimer composed of cytosolic METTL3 and METTL14, followed by ZC3H13mediated METTL3/14 nuclear translocation (Wen et al. 2018a; Liu et al. 2014). In the nucleus, ZC3H13 bridges the METTL3/14 enzymatic core to the MTC chaperone WTAP. The resultant mature complexes are targeted to specific loci through the facultative RNA binding protein (RBP) partners (Wang et al. 2018b; Huang et al. 2019a; Patil et al. 2016). For instance, HAKAI mediates MTC recruitment to the 5’UTR and nascent transcripts near the start codon (Wen et al. 2018a; Liu et al. 2014), VIRMA mediates MTC tethering to the 3'UTR and the stop codon periphery, and RBM15 mediates MTC binding to the U-rich motif with no region preferences. Together, these interchangeable RBPs guide the MTC to deposit m6A at specific regions under different cellular contexts, and thus enhance the operability and flexibility of the m6A machinery.

In addition to the METTL3/14 MTC, other functional complexes catalyze m6A deposition on various RNA species. For instance, the METTL5/TRMT112 and ZCCHC4 complexes catalyze 18S rRNA-1832m6A and 28S rRNA-4220m6A modifications, respectively (Ma et al. 2019); METTL16 catalyzes m6A modification of long non-coding RNAs (lncRNAs) and U6 small nuclear RNA (U6 snRNA) (Pendleton et al. 2017). Overall, tipping the enzymatic balance of the MTC can result in the dysregulation of various biological processes, such as cellular reprogramming (Aguilo et al. 2015), embryonic development (Wang et al. 2014a), and hematopoietic homeostasis (Yao et al. 2018).

### m6A erasers

**Fig. 2:**
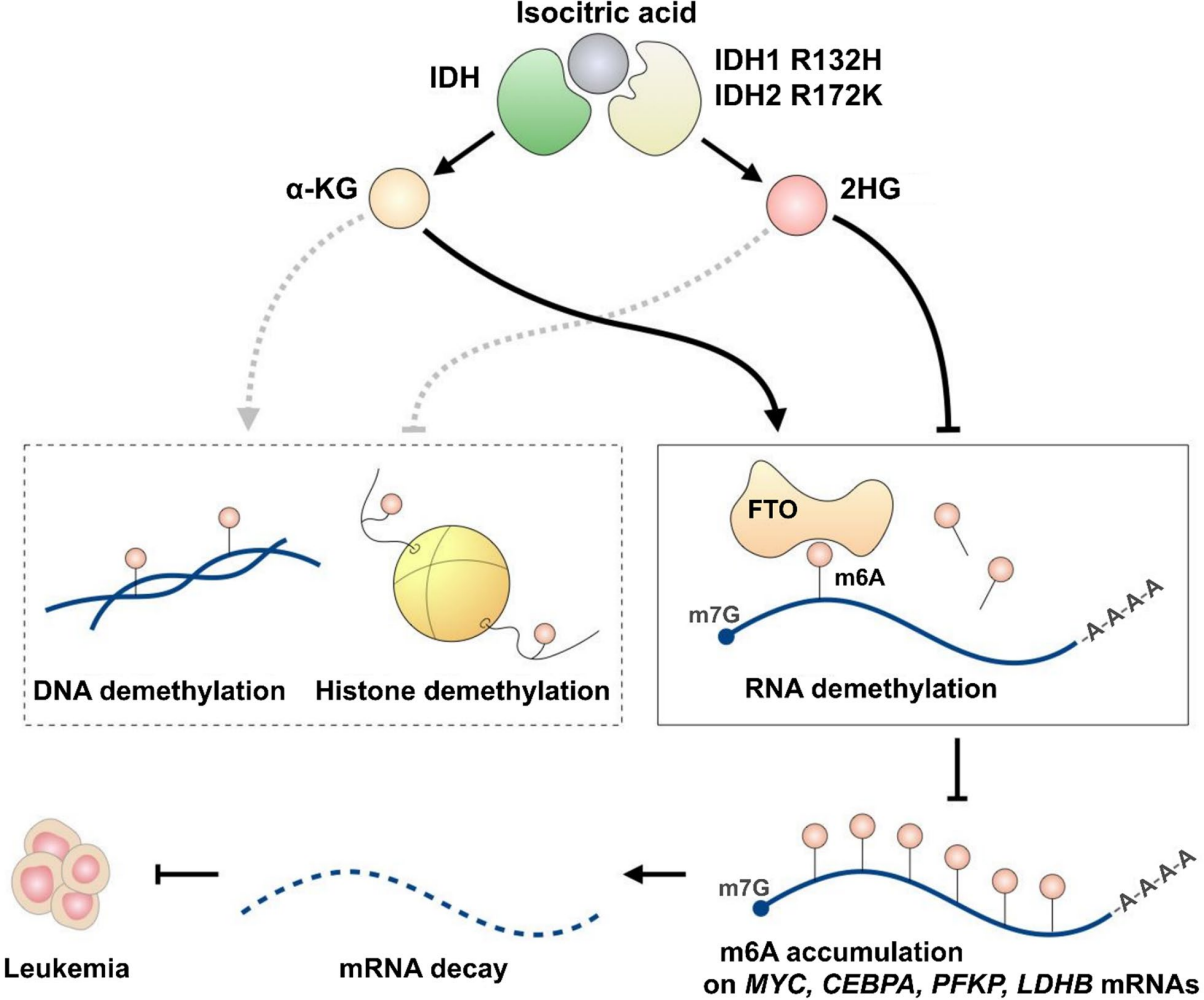
α-KG homeostasis and m6A regulation. In normal conditions, IDH converts isocitric acid to α-KG, which is a cofactor of TET2 demethylase and m6A eraser FTO. Active TET2 participates in DNA demethylation and gene activation. IDH1 R132H and IDH2 R172K mutations produce the 2HG oncometabolite instead of α-KG, which inhibits TET2 and leads to hypermethylated DNA resulting in the downregulation of tumor suppressor gene expression. 2HG production also leads to FTO inactivation resulting in the accumulation of m6A modification on oncogenic mRNAs, promoting their degradation and displaying anti-leukemia potential. In short, 2HG can exhibit both pro-leukemia and anti-leukemia effects through activating distinct pathways

Demethylation of m6A is accomplished by the demethylases FTO and ALKBH5, both of which are dependent on α-ketoglutarate (α-KG) and ferrous ion cofactors. They contain a conserved double-stranded β-helix (DSBH) domain that serves as the catalytic core for the demethylation reaction (Tsujikawa et al. 2007). Indeed, α-KG binds to FTO at R316 and interacts with ALKBH5 via Mn2 + ions on H204, D205, and H266 residues (Feng et al. 2014; Jia et al. 2011). α-KG, a critical intermediate in the tricarboxylic acid (TCA) cycle and amino acid biosynthesis, was first linked to epigenetic mechanisms after being identified as a cofactor of histone and DNA demethylases such as the TET family and the Jumonji domain (JMJD)-containing histone demethylases (Fedeles et al. 2015; Tran et al. 2019). Therefore, reduced α-KG levels abrogate demethylase activity, leading toward the hypermethylated status of DNA, histones, or RNA (Abla et al. 2020; Raffel et al. 2017). Since it is widely known that altered DNA or histone methylation is associated with leukemia (Yang et al. 2019; Wen et al. 2018b), it came as no surprise that α-KG was proven to mediate leukemia progression. For instance, mutations in isocitrate dehydrogenases (IDH), enzymes catalyzing α-KG formation, such as cytosolic IDH1 R132H and mitochondrial IDH2 R172K, disrupt their interaction with the isocitrate substrate and redirect them to the production of hydroxyglutarate (2HG) oncometabolite (Fig. 2) (Ward et al. 2010). Such mutations have been detected in roughly 20% of acute myeloid leukemia (AML) patients. Similarly, D-2-hydroxyglutarate dehydrogenase (D2HGDH) and L-2-hydroxyglutarate dehydrogenase (L2HGDH), the enzymes converting 2HG enantiomers into α-KG, have also been implicated in leukemic transformation. The mutations of D2HGDH and L2HGDH impair the conversion of 2HG isomers into α-KG, causing the accumulation of D-2HG or L-2HG, which inhibits cellular demethylases such as FTO (Wei et al. 2020). Consequently, the accumulation of 2HG in cells inhibits TET2 demethylase, leading to DNA hypermethylation and HIF1α protein degradation (Raffel et al. 2017), eventually promoting leukemogenesis. Interestingly, R-2HG has recently been reported to reduce leukemic progression by inhibiting FTO activity. FTO suppression leads to global accumulation of m6A, inhibiting the pro-oncogenic PFKP/LDHB and MYC/CEBPA signaling axes, ultimately attenuating glycolysis metabolism and inhibiting leukemic cell growth (Su et al. 2018; Qing et al. 2021) (Fig. 2). Therefore, IDH mutants and FTO inhibition possess potential therapeutic values for leukemia treatment by mediating m6A modifications on critical transcripts in leukemogenesis.

### m6A readers

There are three m6A reader families: the YT521-B homolog (YTH) protein family, the heterogeneous nuclear ribonucleoprotein (hnRNP) family, and the common m6A RNA-binding protein family. Different m6A readers subject RNA to enhanced translation or degradation by altering RNA secondary and tertiary structures, as well as by recruiting different effector enzymes.

m6A readers with m6A-recognizing YT521-B homology (YTH) domains are classified into three classes, YTHDC1, YTHDC2, and the YTHDF subfamily (YTHDF1-3). These readers mediate mRNA alternative splicing, nuclear export, degradation, and translation (Wang et al. 2015). Among them, YTHDF2 is the most well-studied, and its canonical mode of action is by selective binding and promoting mRNA decay via recruiting the CCR4-NOT deadenylase complex (Wang et al. 2014b; Du et al. 2016). Functionally, YTHDF2 modulates hematopoietic development by altering the expression of crucial genes, including transcription factors inhibiting B cell to plasma cell transition (*Bach2*, *Pax5*, *Irf8,* and *Spi1*) (Turner et al. 2021), cell cycle regulators in IL-7-induced pro-B cell proliferation (*Zfp87*, *Sertad3*, and *Trib1*) (Zheng et al. 2020), and proinflammatory genes in HSCs (*Stat1*, *Il6r*, and *Gadd45g*) (Mapperley, et al. 2021). In summary, YTHDF2 regulates hematopoiesis by facilitating the degradation of m6A-modified transcripts, thereby decreasing the expression of cell differentiation-related genes. YTHDC1 is a nuclear-residing m6A reader which has a potent effect on embryonic and neural development (Hartmann et al. 1999; Yan et al. 2022). Mechanistically, YTHDC1 regulates RNA alternative splicing via recruiting SRSF3 splicing factor while blocking SRSF10 to promote exon inclusion in target RNAs. Functionally, YTHDC1 mediates the functionality of lncRNAs such as *XIST* and *MALAT1* (Patil et al. 2016; Wang et al. 2021), affecting lncRNA-mediated gene repression. For instance, m6A modification is required for *XIST*-mediated gene suppression and *MALAT1*-induced cell migration and proliferation of esophageal squamous-cell carcinoma (ESCC) cells.

In addition, the hnRNP family members, such as hnRNPC, hnRNPK, and hnRNPA2B1, also recognize m6A sites. The hnRNP family members weakly bind to m6A-modified RNA structures but not directly to m6A itself (Liu et al. 2015), which initiates alternative splicing and translation of target transcripts. Phenitypically, hnRNPs act as adverse prognostic factors in leukemia by licensing oncogenic transcripts for elevated translation, splicing, and stability (Dreyfuss et al. 2002).

Insulin-like growth factor 2 mRNA binding proteins (IGF2BPs) are m6A readers categorized into common m6A RNA binding protein, with emerging role recently in multiple biological pathways, especially in cancer biology. IGF2BP1/2/3 are three proteins in this family, sharing similar structural features. They contain two RNA-recognition motifs (RRMs) and four hnRNP-K homology (KH) domains (Bell et al. 2013). In most cases, IGF2BPs are thought to stabilize the mRNA they bind to and promote their expression (Huang et al. 2018). This regulatory function can be oncogenic, as the expression of a large number of oncogenes in various cancers has been proven to be promoted by this m6A-dependent axis. These targets include VEGF in colon cancer (Yang et al. 2020a), SOX2 in endometrial cancer (Xue et al. 2021), DROSHA and PD-L1 in breast cancer (Peng et al. 2021; Wan et al. 2022), GLUT1 in pancreatic cancer (Huang et al. 2019b), CDK4 in renal cell carcinoma (Gu et al. 2021), just to name a few.

In summary, m6A readers determine the fate of m6A-modified RNA via diverse biological mechanisms and may afterward mediate cellular metabolism or cause tumorigenesis if dysregulated.

## m6A in hematopoietic stem cell maintenance and biogenesis

Hematopoietic stem cells (HSCs) are the progenitors of all differentiated blood cell types. HSCs undergo a dynamic transition between symmetric and asymmetric division to meet the oscillating demands in peripheral blood lineages. Therefore, increasing HSC expansion and optimizing differentiation efficacy are paramount goals in the clinical use of HSCs for transplantation, currently applied to treat conditions such as bone marrow suppression, anemia, and immune deficiency.

Typically, adult HSCs are maintained in a specific environment within the bone marrow, called the niche. The proliferation and maintenance of HSCs are only possible in such a microenvironment, and they lose their ability for self-renewal or undergo cell death outside the niche (Lewis et al. 2021). While adult HSCs residing in the bone marrow are slowly proliferating cells, primitive HSCs arising at the early stages of embryonic development are characterized by an extremely high proliferation rate. At the same time, they are highly resilient to leukemic transformation due to unique characteristics such as increased expression of DNA repair and antioxidant genes (Manesia et al. 2015). Understanding the epigenetic mechanisms governing the self-renewal and proliferation of adult and fetal HSCs, as well as their biogenesis, are of crucial importance for their propagation in vitro for clinical application.

### m6A as a regulator of fetal hematopoiesis

**Fig. 3:**
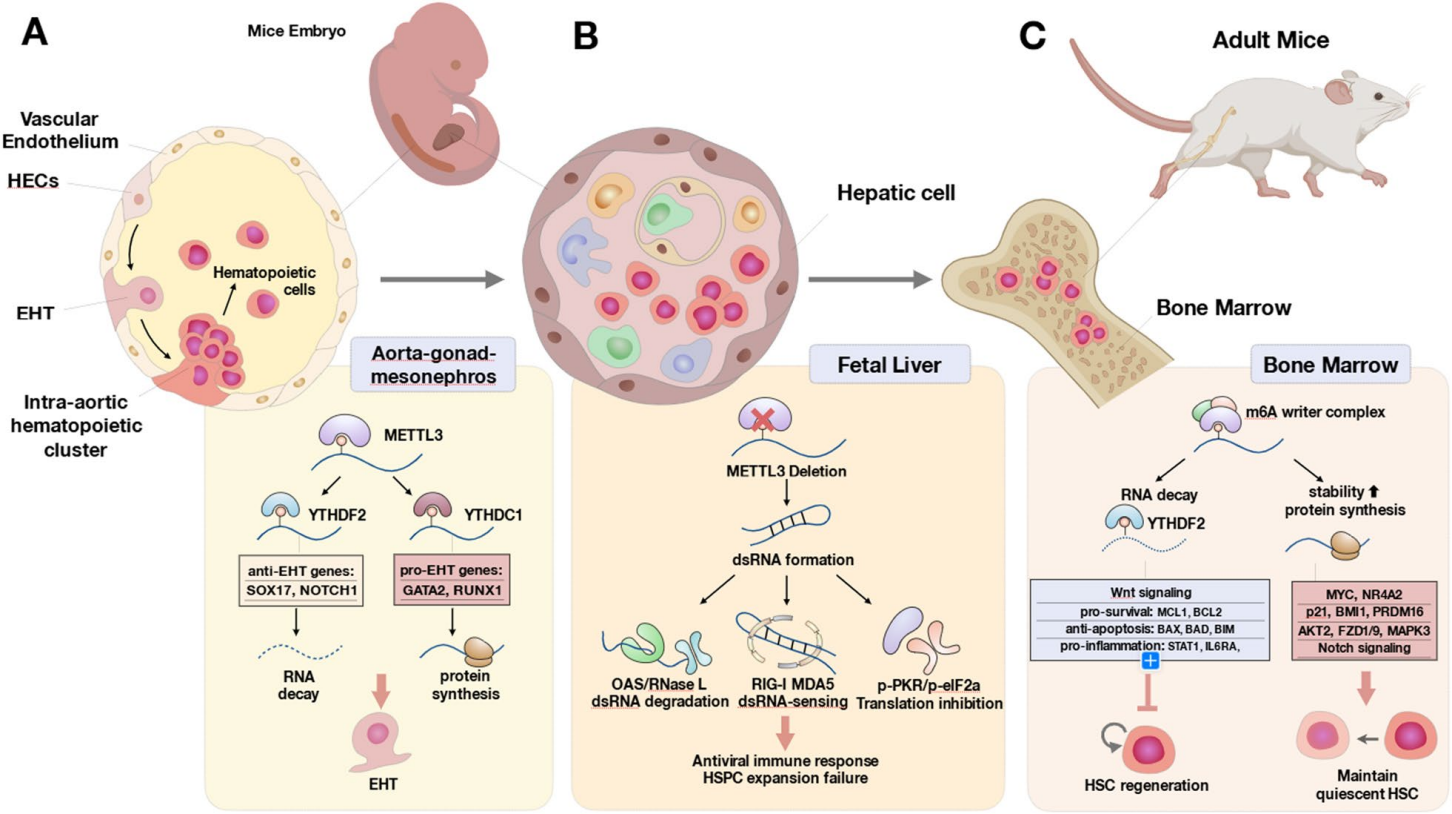
m6A modifications in fetal hematopoiesis and HSC maintenance. **A** Mammalian HSCs are first formed in the aorta-gonad-mesonephros (AGM) region from hemogenic endothelial cells (HECs) as a result of the trans-differentiation process of endothelial-to-hematopoietic transition (EHT). Due to interactions with different readers, m6A-modified transcripts contribute to EHT by promoting pro-EHT, meanwhile suppressing anti-EHT programs. **B** Nascent fetal HSCs migrate to the fetal liver, undergoing rapid and drastic proliferation. (**C**) Adult HSCs are maintained in the niche microenvironment of the bone marrow. The schematics of the principal m6A-regulated pathways occurring at these sites are shown in the bottom panel

During vertebrate embryogenesis, the first HSC population originates from hemogenic endothelial cells (HECs), a specialized type of endothelial cells, in the fetal aorta-gonad-mesonephros (AGM) region and major vessels via a process named endothelial-to-hematopoietic transition (EHT) (Ottersbach 2019) (Fig. 3A). m6A modifications have recently been reported to regulate EHT by altering the expression of several genes encoding pro-hematopoietic factors (GATA2, RUNX1, GFI1, GFI1B, TGFβ, and components of the BMP4-SMAD1/5-HDAC1-ERK axis) (Zhang et al. 2014; Lempereur et al. 2018; Lancrin et al. 2012; Chen et al. 2009), or anti-hematopoietic factors (SOX17 and NOTCH1) (Uenishi et al. 2018; Lizama et al. 2015). For instance, METTL3 suppresses the EHT-inhibiting *notch1a* and *rhoca* mRNAs by facilitating YTHDF2-mediated mRNA decay, which supports HSC production via EHT, as shown in a zebrafish model (Zhang et al. 2017a). *Mettl3* deletion may impair EHT progression by reducing the expression of transcriptional repressors GFI1 and RUNX1, which are the transcription factors essential for HSC development in intra-arterial clusters (Chen et al. 2009; Thambyrajah et al. 2016). GFI1 promotes EHT by binding to the regulatory regions of these genes and recruits LSD1, a chromatin-modifying protein of the CoREST repressive complex, which suppresses gene transcription in HECs of the AGM region during the intra-embryonic wave of hematopoiesis (Thambyrajah et al. 2016). Additionally, RUNX1 enhances the transition of AGM endothelium to hematopoietic cells by regulating the expression of hematopoiesis-specific genes, such as those encoding cytokines IL-3 and GM-CSF; cytokine receptors M-CSFR, B, and T cell receptors, and megakaryocyte-specific chemokine PF4 (Chen et al. 2009; Ichikawa et al. 2013). The HSCs derived from the EHT process then seed the fetal liver, where they further proliferate (Fig. 3B). In hepatic cells, *Mettl3* deletion generates aberrant dsRNA, which activates OAS-RNase L, MDA5-RIG-I, and p-PKR-p-eIF2α axis, eventually leading to innate immune response and hematopoietic failure (Chitrakar, et al. 2021). In this mechanism, OAS detects dsRNA and further activates RNase L, which enhances IFN-β expression and IL-1β activation; RIG-I activates type I interferon transcription while eIF2α decreases global protein translation and promotes the formation of stress granules (Chitrakar, et al. 2021). In summary, recent discoveries have elucidated the role of m6A decoration on EHT-regulating transcripts, providing novel insights into HSC maintenance and offering potential for future stem cell-based therapies in hematopoietic malignancies.

### The role of m6A writers in adult HSC maintenance

Limitations of pharmacology and genetic approaches currently constrain the implementation of HSC-based therapeutics. Therefore, several studies have been conducted to identify whether m6A manipulation could generate novel therapeutic value for HSC-based treatment. Single-cell RNA sequencing (scRNA-seq) of the bone marrow of *Mettl3* conditional knockout (cKO) mice has revealed that m6A loss impairs symmetric HSC division and leads to a decrease in myeloid progenitor production. The m6A-deficient HSCs give rise to a pool of quiescent-free Lin-c-Kit + HSC-like population (Yao et al. 2018) and are blocked from entering myeloid progenitor checkpoints. In line with this finding, another *Mettl3*-deficient mice study showed reduced *Nr4a2*, *Cdkn1a* (p21), *Bmi1*, and *Prdm16* expression concomitant with HSC accumulation (Yao et al. 2018) (Fig. 3C). Interestingly, a transgenic mice study using lineage-specific *Mx1-cre; Mettl3fl/fl* (induced *Mettl3* KO at HSC stage) and *Lysm-cre; Mettl3fl/fl* (induced *Mettl3* KO at myeloid progenitor stage) plpC induction system revealed that *Mettl3* is essential to maintain HSC properties, whereas it is redundant or dispensable for myeloid progenitors to generate functional macrophage phenotype. Therefore, this evidence strongly implies that the demand for m6A epitranscriptome is context-dependent and stage-specific.

Other m6A writer complex members (WTAP and RBM15) are also crucial for HSC differentiation. Through poly (I:C) inducible *Wtap*^*fl/fl*^*-Mx1-Cre* mouse model, *Wtap* deletion led to twice the average pool size of HSCs and LSKs, progenitor counts of HPCs, CLPs, and LMPPs were also expanded (Liu et al. 2020). The deletion of *Wtap* could reduce the expression of pluripotency-associated genes such as *Akt2*, *Fzd1/9*, and *Mapk3* (Liu et al. 2020). Also, the depletion of RBM15 affected downstream hematopoiesis, such as blocking B cell differentiation (Raffel et al. 2007), and suppressing myeloid differentiation through the NOTCH signaling pathway (Raffel et al. 2007). In short, m6A writers (including METTL3, METTL14, WTAP, and RBM15) regulate HSCs quiescence and hematopoiesis in an m6A-dependent manner.

### m6A reader proteins in HSC regulation

In light of the in vitro and in vivo HSC *Mettl3* KO results, parallel experiments were repeated with the KO of m6A reader proteins, namely the YTH domain and IGF2BP m6A RBP families. While *Mettl3* KO models linked decreased m6A deposition to HSC expansion and differentiation blockade (Cheng et al. 2019), several *Ythdf2* KO studies showed that abrogated m6A-mediated mRNA decay also led to phenotypic HSC expansion (Li et al. 2018). Wang et al. further demonstrated that *Ythdf2*-null HSCs, in comparison to wild-type HSCs, exhibited a 50% increment of the long-term repopulating ability in competitive transplantation models (Wang et al. 2018a). On top of expanded HSC quantity, *Ythdf2*^−/−^ HSCs showed no homing deficiency during the study interval and no defects in reconstituting multilineage hematopoiesis (Mapperley et al. 2021). Moreover, the regenerative capacity of *Ythdf2*^−/−^ HSC transplants was higher than that of the *Ythdf2*^+/+^ post-5-FU and radiation bone marrow resetting treatment. These data indicate that *Ythdf2* KO, just like the *Mettl3* KO approach, can be applied to potentiate the resilience and robustness of the HSCs. Nevertheless, a longitudinal study demonstrated that *Ythdf2*-null HSCs harvested from young mice eventually fail throughout serial transplantations. In the late-propagated generations of *Ythdf2*^−/−^ HSCs(Mapperley et al. 2021), chronic inflammation was documented, in which the m6A-modified transcripts transcribed under pro-inflammatory IFN-γ, TNF-α, STAT1, IRF7, and TLR4 programs were accumulated, a consequence attributed to the absence of YTHDF2-mediated mRNA decay. Therefore, the *Ythdf2* KO seemingly causes HSC expansion at its imminent phase but results in an inflammatory failure at its delayed stage.

*Ythdf3* and *Ythdf1* are two conservative m6A reader homologs of *Ythdf2*. Nevertheless, only *Ythdf3* KO generated a defective hemogram, whereas *Ythdf1* KO resulted in a regular profile in HSC progenitors and differentiated peripheral blood cells (Zhang et al. 2022). Furthermore, in the competitive transplantation assay, the *Ythdf3* KO HSCs showed impaired reconstitution of T cell, B cell, and myeloid lineages as early as 3 months post-transplantation (Zhang et al. 2022). In the subsequent experiments, full transcriptome analysis revealed alterations in ribosome and protein synthesis pathways in *Ythdf3* KO cells (Zhang et al. 2022). Through click chemistry-based analysis of total translation, *Ythdf3* KO resulted in a reduced flux of protein synthesis, thus linking replication stress to HSC stasis. Although the previous *Ythdf2* KO experiment identified six m6A-regulated HSC genes (*Myc*, *Ccnd1*, *Axin2*, *Mcl1*, *Cd133,* and *Bcl2*) in LSK cells, *Ccnd1* (cyclin D1) was the only gene verified to be suppressed in *Ythdf3* KO HSCs (Wang et al. 2018a; Li et al. 2018). Mechanistically, *Ccnd1* translation was abrogated when *Ythdf3* KO led to destabilized PABPC1-EIF4G2 complex and disrupted its binding to *Ccnd1* 5’UTR, thus resulting in decreased translation initiation (Zhang et al. 2022). The overall *Ythdf3* KO-mediated *Ccnd1* regulation in HSC failure was not reproducible by *Ythdf1* KO. From this point, we conclude that among three *Ythdf* homologs, *Ythdf1* is less contributive to HSC regulation. At the same time, *Ythdf2* and *Ythdf3* KO affect HSC biology through transcript degradation and transcript translation (Du et al. 2016; Shi et al. 2017), respectively.

As supported by preclinical results, m6A and its regulators were proposed to be potentially used for HSC-based therapeutic applications. For instance, it has been shown that YTHDF2 suppresses pro-inflammatory transcripts such as *Stat1*, *Il6ra,* and *Gadd45g* in the HSCs (Mapperley, et al. 2021), thereby preventing chronic HSC inflammatory status. On the other hand, YTHDF2 also restrains the overt proliferation of HSCs by degrading the m6A-labeled transcripts encoding components of the Wnt signaling axis (*Myc*, *Ccnd1*, *Axin2*) (Wang et al. 2018a), pro-survival pathways (*Mcl1*, *Bcl2*, *Bax*, *Bad,* and *Bim*) (Morales et al. 2011), as well as *Tal1*, encoding a critical important transcription factor for hematopoiesis (Wang et al. 2018a; Li et al. 2018). Collectively, HSCs employ m6A RNA modifications as an epigenetic switch, by which the subject gene sets can be turned on or off through context-dependent m6A labeling. Consequently, approaching transcriptome balance and HSC homeostasis.

## The role of m6A in normal lymphoid hematopoiesis

### The role of m6A in innate lymphoid cells (ILCs): natural killer (NK) cells, ILC1, ILC2, & ILC3

**Fig. 4:**
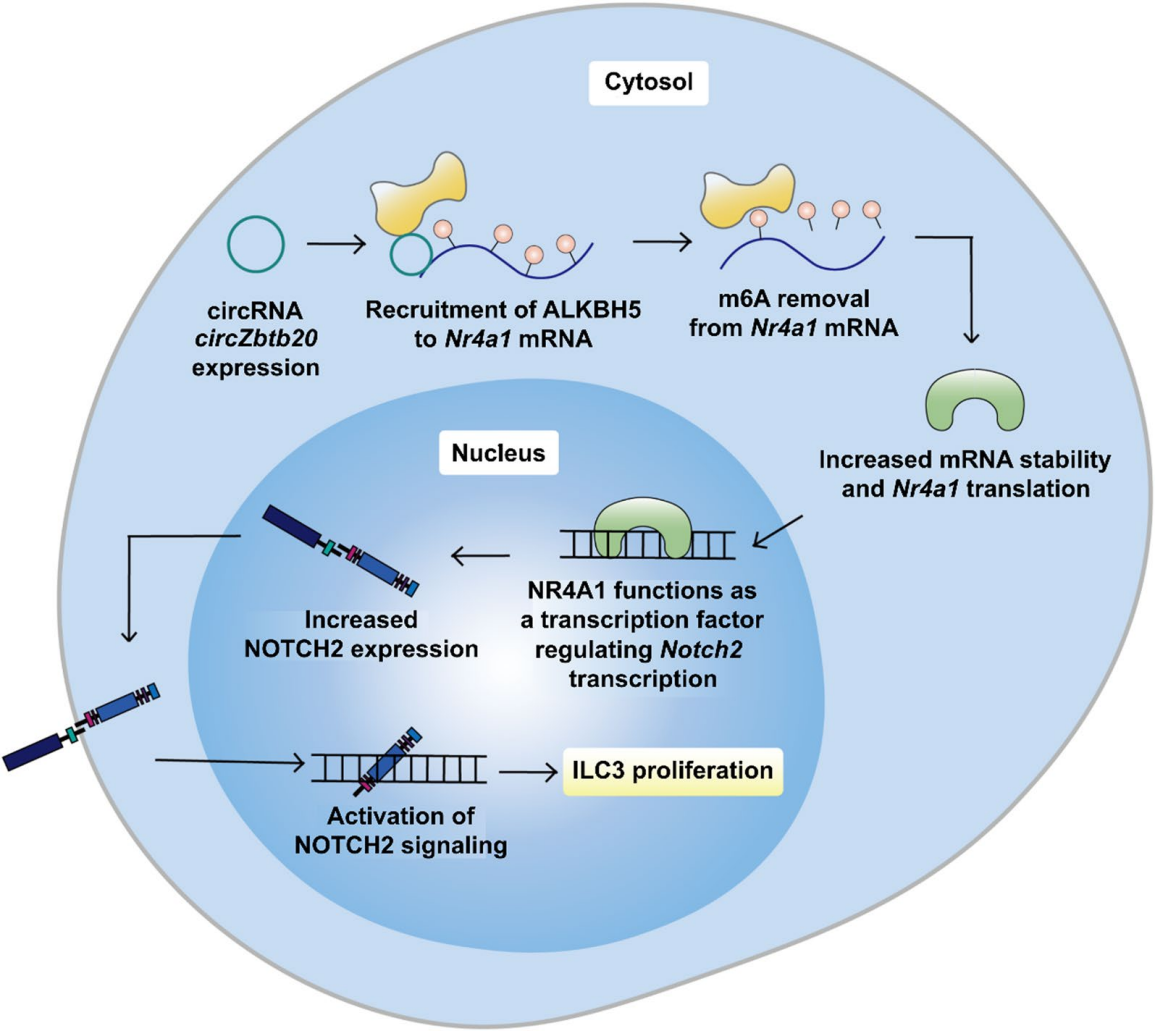
Promotion of ILC3 cell proliferation in m6A-dependent manner. Circular RNA *circZbtb20* promotes m6A removal from *Nr4a1* mRNA by recruiting m6A eraser ALKBH5. This prevents YTHDF2-dependent degradation of *Nr4a1* mRNA and thus increases the expression of NR4A1 transcription factor. NR4A1 activates the NOTCH2 signaling pathway that promotes ILC3 cell proliferation

Innate lymphoid cells (ILCs) comprise ILC1/2/3, natural killer (NK) cells, and lymphoid tissue inducer (LTi) cells. ILCs are differentiated from the common lymphoid progenitors (CLPs) and participate in the immune responses at the epithelial barrier surface when activated by signals, such as cytokines, in their immediate environment (Iwafuchi et al. 2016). Although ILC1/2/3 displays lymphoid morphology like NK or B cells, they do not express T cell receptors (TCRs) or undergo genetic rearrangement during maturation (Mjösberg and Spits 2016; Morita et al. 2016). Notably, m6A regulators mediate the expression of critical transcription factors during ILC differentiation and commitment. For instance, METTL3 promotes T-bet expression required for ILC1 development and the RORγT/Notch signaling in ILC3 development (Yao et al. 2021; Possot et al. 2011); KIAA1429 prevents transcript decay of GATA3, a transcription factor critical for ILC2 commitment (Lan et al. 2019; Kasal et al. 2021). Intriguingly, ILC behavior is also regulated by a crosstalk between ncRNAs and m6A: circRNA *circZbtb20* facilitates ALKBH5-mediated m6A removal on *Nr4a1* mRNA, contributing to the maintenance of ILC3 proliferation ability through enhancing NOTCH2 signaling (Liu et al. 2021a) (Fig. 4). To conclude, growing evidence demonstrates a robust role of m6A in ILC behavior. Given that ILC functions are associated with leukemic progression (Trabanelli et al. 2017), further investigation of the role of m6A-decorated transcripts in ILC regulation is required to develop promising immunotherapeutic strategies targeting the tumor microenvironment.

Among ILCs, NK cells target aberrant autologous cells, such as virus-infected cells, and malignantly transformed cells by recognizing MHC class I molecules (Hansen and Bouvier 2009). Anti-leukemia immunity of NK cells is stimulated mainly through the IL-2 or IL-15-STAT5 pathways (Szczepanski et al. 2010; Lehmann et al. 2001), which have recently been linked to the m6A reader YTHDF2. YTHDF2 forms a positive feedback loop with STAT5 in response to IL-15 stimulation. Such loop promotes production of IFN-γ, granzyme B, and perforin, subsequently enhancing NK cell activation and cytotoxicity (Ma et al. 2021). In addition, YTHDF2 was found to be essential for NK cell growth and activation by enhancing the degradation of *Tardbp* mRNA, the newly identified YTHDF2 target (Ma et al. 2021). Therefore, YTHDF2 deficiency resulted in reduced NK cell count and abrogated cytotoxicity.

Similarly, METTL3 is also essential for NK cell maturation and expansion via m6A decoration. A recent study showed that METTL3 targets and enhances the expression of SHP-2 tyrosine phosphatase (encoded by *Ptpn11*), activating the AKT-mTOR and MAPK-ERK pathways upon IL-15 overexpression, eventually sustaining cytotoxic molecules production. Moreover, in *Mettl3* KO mice, a cell marker specific for terminally differentiated NK cells termed killer cell lectin-like receptor G1 (KLRG1), was found to be downregulated in the spleen, lung, liver, and bone marrow (Song et al. 2021). Overall, METTL3 deficiency alters the m6A landscape in critical transcripts, resulting in reduced NK cell infiltration in the tumor microenvironment, decreased sensitivity to IL-15 overexpression, and impaired clonal expansion in several peripheral organs.

### The role of m6A in T cell biogenesis

m6A regulators regulate T cell differentiation, proliferation, and activation. For instance, *Mettl14* cKO was shown to increase the population of inflammatory T cells (such as Th1 and Th17) while suppressing differentiation towards regulatory T cells (Tregs) (Lu et al. 2020). In addition, *Mettl3* deficiency blocks the differentiation of naive T cells into effector T progenitors in mice (Li et al. 2017). m6A epitranscriptome controls the suppressor of cytokine signaling (SOCS) protein family (including SOCS1-3 and CISH) in T cell biology via the METTL3-YTHDF2-SOCS axis, in which the SOCS proteins inhibit IL-7 and STAT5 and block T cell signaling cascade (Palmer and Restifo 2009). On the post-transcriptional level, SOCS transcripts can be methylated by METTL3 and targeted for degradation by m6A-YTHDF2 binding. The m6A-mediated SOCS silencing was found to lead to IL-2/STAT5/FOXP3 activation, which resulted in Treg differentiation and expansion (Yao et al. 2021), while the IL-7/STAT5/SOCS pathway facilitated naive T cell reprogramming and proliferation (Wu et al. 2019). In short, the SOCS protein family is an important m6A downstream effector in T cells.

In addition, m6A affects T follicular helper (Tfh) cell differentiation through the VHL/HIF-1α/GAPDH/ICOS axis (Zhu et al. 2019). Furthermore, METTL3 is indispensable for the expression of m6A-modifiable Tfh signature transcripts (*Bcl6*, *Tcf7*, and *Cxcr5*) (Yao et al. 2021) whereas the anti-Tfh differentiation genes (*Foxo1*, *Prdm1*, and *Tbx21*) (Zhu et al. 2019) were inhibited by METLL3. In summary, the m6A modification network modulates cytokine production and transcription programming to direct T cell fate.

### The role of m6A in B cell biogenesis

B cells are responsible for the adaptive immune response, in which a diverse B cell receptor (BCR) repertoire is required to bind antigens. BCRs are generated through genetic recombination, diversified through class switch recombination (CSR) and somatic hypermutation (SHM), all requiring multistep coordination of transcriptome programming. Among them, in some incidences, the remodeling of B cell transcriptomes is tightly regulated by the m6A machinery. Hence in the following sections, we will summarize the crosstalk between m6A regulators and B cell immunity.

According to the immunoglobulin (V, D, and J segment of H chain, and V, J segment of L chain) rearrangement status, B cell development in bone marrow can be divided into four stages: pro-B cell, large pre-B cell, small pre-B cell, and immature B cell (Pieper et al. 2013). In such a context, *Mettl14* KO impaired IL-7-induced pro-B cell proliferation and its maturation into the large pre-B stage by disturbing YTHDF2-modulated mRNA decay and causing dysregulation of cell cycle and BCR recombination-related genes. METTL14 is essential for transitioning large pre-B cells to small pre-B cells in the subsequent differentiation stage. It mediates the appropriate transcription by interacting with transcription factors through the process independent of YTHDF1/YTHDF2 (Zheng et al. 2020).

After leaving the bone marrow, immature B cells are transferred to the spleen to further mature into transitional T1 and T2 B cells (Loder et al. 1999). T2 B cells differentiate into the follicular and marginal zone (MZ) B cells. Among them, follicular B cells form or enter the germinal center (GC) (Pieper et al. 2013). When constructing and maintaining a GC, B cells require the m6A-binding protein IGF2BP3 to stabilize the mRNAs of genes responsible for the proliferation downstream of Myc. Besides, another m6A reader, YTHDF2, is necessary to properly function the electron transport chain in the mitochondria of GC B cells (Grenov et al. 2021).

When B cells encounter antigens, the elevated activation-induced cytidine deaminase (AID) expression cooperates with activated T cells to clear antigens in the GCs. GCs are the structures in the B cell zone of lymph nodes, where B cells later undergo class switching recombination (CSR) and somatic hypermutation (SHM) and differentiate into antibody-secreting plasma cells and memory B cells (Schmidlin et al. 2009). Nascent-transcribed ncRNA SμGLT forms an R-loop structure at the IgH locus, and m6A modification of SμGLT promotes its recognition by YTHDF1. YTHDF1 stabilizes the R-loop structure and synergistically works with MPP6 adaptor protein to recruit AID DNA deaminase and RNA exosome, which CSR requires. Therefore, inhibiting YTHDF1 or suppressing m6A leads to decreased DNA-RNA hybrid R-loop structure and decreased AID recruitment, reducing the efficiency of B cells conducting CSR (Nair et al. 2021).

B cells in GC can differentiate into plasma cells and memory cells (Klein and Dalla-Favera 2008). It was observed that METTL14-deficient mice are incapable of eliciting proper GC B cell response, including proliferation and SHM deficiency. Knocking out *Mettl14* downregulates genes related to cell cycle G2/M transition regulation and GC B cell positive selection through YTHDF2-dependent mRNA degradation. METTL14 can also decrease the expression of negative regulators, such as *Lax1* and *Tipe2*, in positively selected GC B cells (Huang et al. 2022). In summary, m6A plays a vital role in crucial branching steps of B cell differentiation, and how m6A acts in B cell life history is still to be discovered.

## The role of m6A in lymphocytic hematopoietic malignancies

Lymphocytic hematopoietic malignancies are a group of neoplasms stemming from uncontrolled proliferated and aberrant differentiation of lymphoid precursors, including lymphocytic leukemia and lymphoma. Numerous factors contribute to the initiation, progression, and prognosis of this type of malignancies, including damaged gene regulation, impaired cell metabolism, and dysregulated epigenetic networks. The roles of RNA methylation in lymphocytic hematological cancers were found to be increasingly crucial. Therefore, in this section, we discuss the regulation of the m6A RNA methylation landscape in different RNAs (mRNAs, ncRNAs, and rRNAs) in lymphocytic leukemia and lymphoma and dissect the molecular characteristics of cells with dysregulated m6A methylome.

### Acute lymphoblastic leukemia and chronic lymphocytic leukemia

Acute lymphoblastic leukemia (ALL) and chronic lymphocytic leukemia (CLL) result from abnormal lymphoid differentiation. ALL blasts are transformed from less mature lymphoid precursors than CLL blasts, thus leading to more rapid progression of the former. Typically, m6A enzymes regulate the expression homeostasis of oncogenes and tumor suppressor genes to promote or inhibit leukemogenesis, while leukemia cells often display an altered m6A methylation landscape.

To begin with, METTL3 enhances cell survival in CLL by enhancing leukemic gene translation (*SF3A1*, *SF3A2*, *SF3B1*, *U2AF1*) (Wu et al. 2020; Fei et al. 2018; Zhang et al. 2017b; Rozovski et al. 2013). In T-cell ALL (T-ALL) and *MLL*-rearranged (*MLL*-r) ALL, METTL16 was shown to stimulate the production of *MAT2A* mRNA encoding methionine adenosyltransferase 2A (MAT2A, the SAM synthase) by promoting the splicing of an intron, which afterward enables the functions of multiple methyltransferases, including DOT1L and PRMT5 (Pendleton et al. 2017; Secker et al. 2020), thereby provoking pathogenesis. Similarly, IGF2BPs were reported to promote leukemia progression by maintaining the stability of m6A-labeled oncogenic mRNAs encoding self-renewal regulators HOXB4 and MYB, aldehyde dehydrogenase ALDH1A1 (Elcheva et al. 2020), the stem cell reprogramming factor LIN28B (which downregulates let-7 miRNA) (Zhou et al. 2017), and c-MYC (Zhu et al. 2021). Notably, the m6A-reading IGF2BP family also plays important roles in ALLs with distinct genetic signatures (Stoskus et al. 2011). IGF2BP1 overexpression targets critical genes, such as the *ETV6*/*RUNX1*-*RAC1*-*STAT3*-*MYC* axis (Stoskus et al. 2016) and the TNFα/NFκB signaling (*IL6ST*, *NFAT5*, *CDK6*, *MDM2*, *CCND1*, *NGFR*) (Sharma et al. 2021). The process is possibly the “second hit” of leukemia progression after a particular chromosomal rearrangement, the t(12;21)(p13;q22) *ETV6*/*RUNX1* or the t(14;17)(q32;q21) *IGH*/*IGF2BP1* translocation in childhood B-ALL (Gu et al. 2014; Palanichamy et al. 2019), therefore, it enhances its cellular survival, self-renewal, and proliferation. Correspondingly, IGF2BP3 is commonly overexpressed in *MLL*-r B-ALL and Hodgkin lymphoma and supports tumorigenesis via promoting mRNA stability of *MYC* and *CDK6* (Palanichamy et al. 2016; Masoud et al. 2019). By studying different ALL patterns with gene translocations, better management of the disease could be achieved in the future. In short, these m6A regulators which appear as oncogenes are often overexpressed during lymphoid leukemias development and modulate transcripts promoting cell survival and proliferation.

Despite the evidence for the association between m6A and leukemogenesis, ALLs with inheritable gene rearrangements demonstrated contradictory results. For example, lower *METTL3* and *METTL14* expression levels were associated with the progression of pediatric *ETV6*/*RUNX1*-positive ALL and a higher relapse rate (Sun et al. 2019; Liu et al. 2021b; Luo et al. 2021). These results indicate that the roles of METTL3 and METTL14 may vary in ALLs with different genetic mutation patterns. Besides mRNA, m6A inhibits leukemogenesis by regulating rRNA as well. For instance, METTL5, the m6A methyltransferase of rRNA, methylates 18S rRNA at position 1832A, which results in the proper folding of the ribosome decoding center. This methylation promotes the translation of tumor suppressor genes such as *FBXW7*, *KLF4*, *SOX2*, and *REX1*, thus antagonizing leukemogenesis. Moreover, *Mettl5* KO promotes leukemia growth by translationally inhibiting F-box and WD repeat domain-containing 7 (*Fbxw7*) (Xing et al. 2020; Yeh et al. 2018; King et al. 2013), a c-MYC degrader and a key regulator of cell differentiation. Therefore, decreased FBXW7 leads to c-MYC accumulation and NOTCH activation essential for cell survival and proliferation of chronic myeloid leukemia (CML) and B-cell ALL (B-ALL) (Reavie et al. 2013).

Our current understanding of targeting m6A regulators has generated potential therapeutic targets. For instance, FB23-2 is an FTO inhibitor developed as a candidate drug for treating leukemia. This compound forms hydrophobic interactions with FTO’s nucleotide recognition lid motif of FTO and abrogates its demethylation function. FB23-2 treatment promotes myeloid cell differentiation and primes leukemic cells for a p53-mediated apoptotic program by abrogating c-MYC and CEBPA anti-apoptotic proteins (Huang et al. 2019c). On the other hand, ALKBH5 enhances enzyme expression along the USP1-Aurora B axis in T-ALL by demethylating m6As at the 3'-UTRs of target transcripts (Gong et al. 2021), increasing mRNA stability and promoting cancer progression. Therefore, shALKBH5 significantly ablates AML growth with little effect on normal hematopoiesis, suggesting potential therapeutic roles of ALKBH5 inhibitors (Shen et al. 2020; Selberg et al. 2021). In brief, the m6A methylome participates in the hematopoietic process and leukemia growth and is engaging in identifying novel therapeutic targets for blood cancer.

### The role m6A in lymphomas

Cancer cells circulate throughout the body in lymphoblastic leukemias, while lymphoma cells tend to aggregate and form neoplasms in the bone marrow. There are two main subtypes of lymphoma: Hodgkin lymphoma (HL) and non-Hodgkin lymphoma (NHL). Diffuse large B-cell lymphoma (DLBCL) is the most common category of NHL with abnormally large B lymphocytes and fast-growing speed. Both subtypes show aberrant m6A methylation traits. For example, METTL3 stimulates tumor proliferation of DLBCL through PEDF-mediated Wnt/β-catenin apoptotic signaling repression (Cheng et al. 2020; Ma et al. 2017). Furthermore, WTAP also plays an oncogenic role in DLBCL. In the presence of piRNA-30473, the increased WTAP level promotes the expression of the oncogenic *HK2* gene by inducing IGF2BP-mediated transcript stabilization in an m6A- dependent manner (Han et al. 2021). Moreover, the expression of the YTHDF2 reader was found to be associated with poor DLBCL prognosis by targeting the ACER2-ceramide metabolic axis (Dixit et al. 2021). Additionally, the overexpression of the m6A-binding protein hnRNPK improves ribosome loading efficiency to the *Myc* transcript (Evans et al. 2003), thus increasing c-MYC protein level and causing DLBCL propagation (Gallardo et al. 2020).

The prognosis of DLBCL is also affected by the crosstalk between m6A regulators and m6A-modified lncRNAs, including *TRERNA1* and *NBAT1* (Li et al. 2022; Wei et al. 2021; Song et al. 2022). ALKBH5 upregulates the expression of *TRERNA1* via m6A removal, thereby promoting the *TRERNA1*-EZH2 interaction and repressing the *CDKN1A* (p21) promoter region. This inhibits the expression of the cell cycle inhibitor p21, eventually leading to poor prognosis in DLBCL (Song et al. 2022). Another lncRNA, *NBAT1*, blocks the interaction of IGF2BP1 and *MYC* transcripts to destabilize *MYC* mRNA, and inhibits leukemic growth (Li et al. 2022; Wei et al. 2021). To conclude, multiple m6A targets cooperate to regulate cell transformation and proliferation in lymphoma, leading to pessimistic disease outcomes. These biomarkers may become predictive or diagnostic tools and serve as novel therapeutic targets for lymphoid hematological cancers in the future.

## A case of lncRNA: interplay between m6A and global genome regulators

The functionality of eukaryotic organisms relies on the relatively limited number of protein-coding genes, and the level of complexity of such organisms does not necessarily correlate with their number. On the other hand, the astounding complexity of higher eukaryotes’ organization relies on multiple layers and complex networks of epigenetic regulation that ensure highly coordinated expression of the protein-coding gene toolbox. m6A modification of mRNA represents one of such epigenetic layers. In this review, we summarized its role in coordinating the expression of specific protein-coding genes in the immune cell hematopoiesis and dysregulation in associated malignancies on the mRNA level. However, different layers of epigenetic regulation are highly interconnected in a complex manner.

Interestingly, global epigenetic regulation of the genome often relies on RNA-based regulators, which can be the direct targets of m6A modification, thus linking the epitranscriptomic level with epigenetics. lncRNAs represent the majority of transcriptional output of the genome, and many of them were characterized as global chromatin structure regulators. X-inactive specific transcript (*XIST*) is one such global regulator lncRNAs, as it is essential for gene dosage compensation by silencing one of two X chromosomes. It has also been implicated in lymphoid biology in an m6A-dependent manner. Here, we summarize the role of m6A modifications of *XIST* lncRNA in various aspects of lymphoid biology.

*XIST* is a 17 kb lncRNA required for inducing X chromosome inactivation in female placental mammals by wrapping around the entire chromosome and recruiting gene silencing complexes such as polycomb and NCOR/HDAC3 (Avner and Heard 2001). This process is mediated by m6A modification of *XIST* in a context-dependent manner. The METTL14-YTHDF2 pathway initiates *XIST* degradation, while the METTL3-YTHDC1 axis promotes *XIST*-mediated transcriptional repression (Patil et al. 2016; Yang et al. 2020b). Once the adenosine of AUCG tetraloop on *XIST* is methylated by the METTL3-RBM15-WTAP complex, it forms a hairpin structure at its A-repeat region (Patil et al. 2016; Jones et al. 2022). Such folding of *XIST* is required to bind m6A reader YTHDC1, and the resultant RBP initiates X chromosome condensation and gene silencing (Jones et al. 2022; Syrett et al. 2017).

**Fig. 5:**
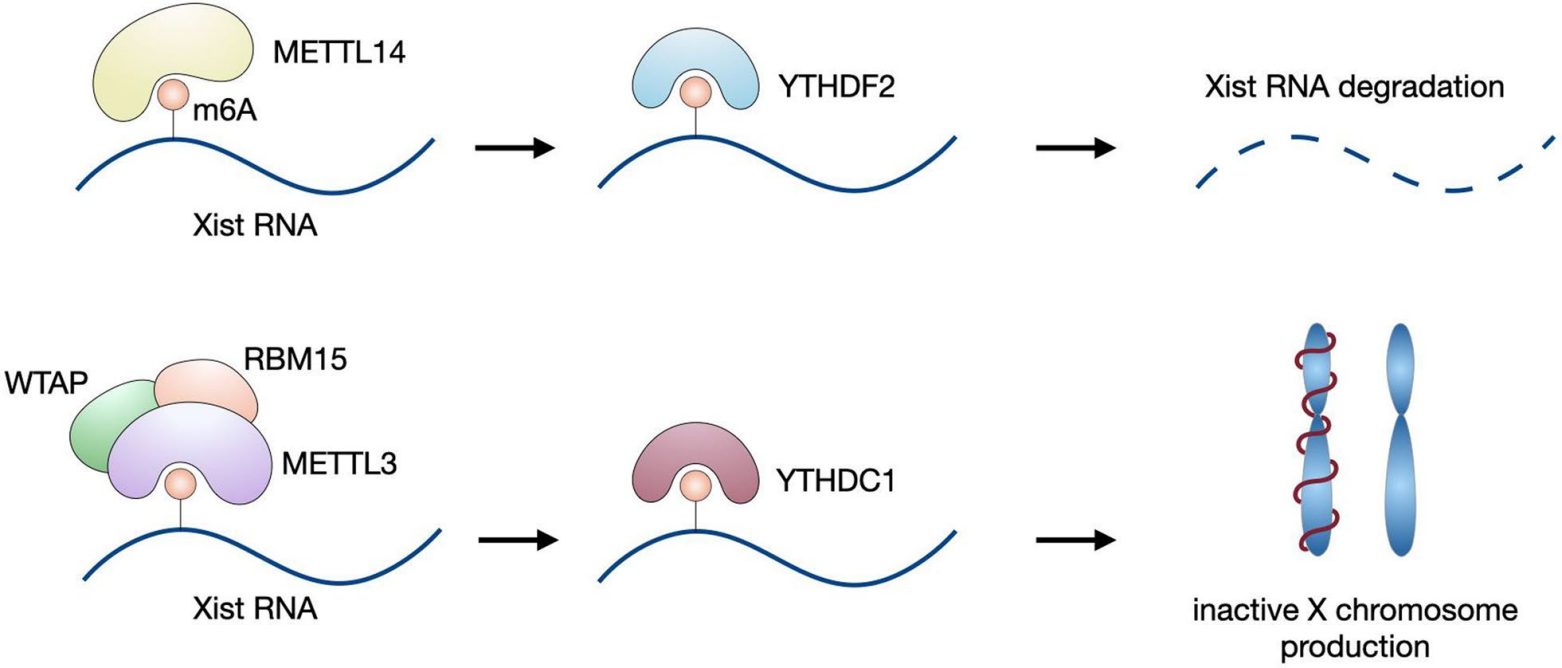
Function of m6A on *XIST* lncRNA. The downstream effect of m6A on Xist RNA depends on the reader protein. When YTHDF2 interacts with m6A-modified *XIST*, it promotes its degradation; however, when the m6A/YTHDC1 axis is dominant, gene silencing machinery is turned on and inactive X chromosomes are produced

Dysregulated m6A modifications on *XIST* correlate with autoimmune disease progression, such as systemic lupus erythematosus (SLE) and rheumatoid arthritis (RA). In the absence of *XIST*, multiple *XIST*-regulated immune genes (e.g., *TLR7*, *IRAK1*, *XIAP*, *TSC22D3*, and *MMP1*) are overexpressed, resulting in the formation and expansion of CD11c + atypical memory B cells (ABCs, a unique B cell population indicating the onset of aging, infection, SLE or RA) (Yu et al. 2021; Karnell et al. 2017; Cancro 2020; Woodruff et al. 2020), B cell autoantibody production (Pyfrom et al. 2021), and overexpression of *XIST*-regulated genes during T cell maturation of SLE patients and mice, eventually contributing to SLE pathogenesis (Syrett et al. 2019). This evidence indicates the critical role of m6A and *XIST*-related mechanisms in autoimmune diseases. Meanwhile, the m6A modification of *XIST* also plays an oncogenic role in multiple solid cancers, such as colorectal and breast carcinomas. In summary, m6A affects the immune cell development status (Syrett et al. 2017), autoimmune disease pathology, and cancer progression by enhancing the expression of *XIST*-regulated immune genes (Fig. 5).

## Conclusions

**Fig. 6:**
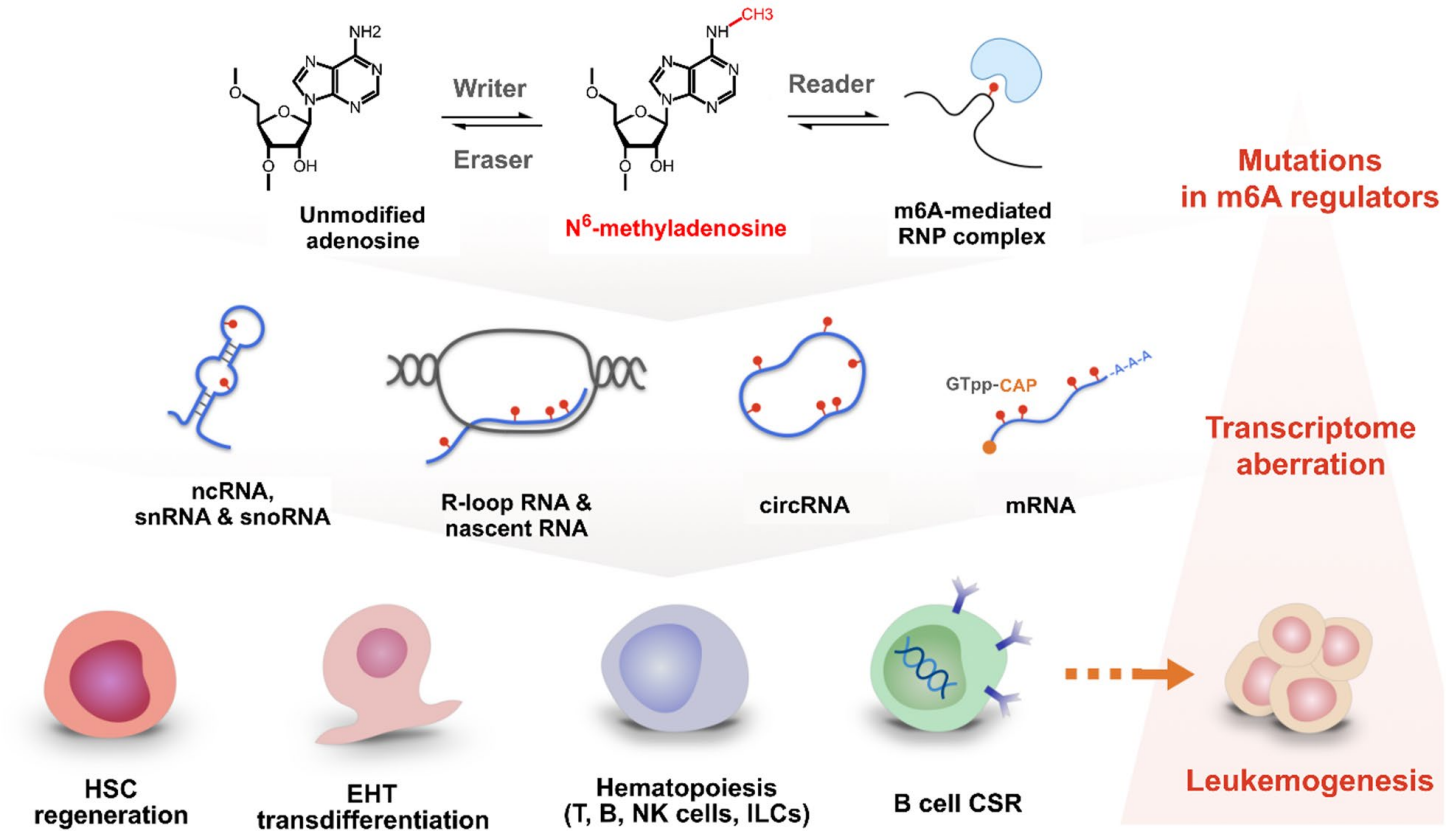
Overview of m6A-dependent mechanisms in lymphoid system-related processes. From top to bottom: overview of m6A regulation machinery, typical substrates, and processes. Right panel: pathological events can start from mutations in m6A regulators which lead to transcriptome aberration and development of leukemias or lymphomas

Collectively, the m6A epi-transcriptome governs the self-renewal and lineage commitment in hematopoietic biology. The m6A modification primes cellular events such as in hemogenic endothelial EHT, T cell differentiation skewness, B cell CSR/SHM, ILC immunity, and oncometabolite mediated leukemogenesis. At the transcript level, m6A also regulates RNA biophysical properties by orchestrating mRNA turnover, RNA binding protein affinity, and XIST mediated gene silencing (Fig. 6, Table 2).

**Table 2:** Summary of the key regulators of m6A system in the hematopoiesis processes and diseases

m6A regulator	Subjects	Cellular effect of m6A regulator knockdown/depletion				Mechanism
		Differentiation	Regeneration	Apoptosis	Others	
m6A writer						
METTL3 (Lv et al. 2018)	mHSCs				↓ EHT	↓METTL3/YTHDF2/↑notch1a
METTL3 (Yao et al. 2021)	mice Tfh cells	↓				↓Mettl3/↓Tcf7, Bcl6, Icos and Cxcr5
METTL3 (Li et al. 2017)	mice T cells					↓METTL3/↑Socs1, Socs3 and Cish/↓IL-7–STAT5 signaling; ↑ERK & AKT signaling
METTL3 (Nair et al. 2021)	human B cells				↓ CSR	↓SμGLT/↓YTHDC1 & MPP6/↓CSR
METTL3 (Choe et al. 2018)	human cells (e.g. HEK293T and HeLa)				↓ oncogene (BRD4) translation	↑METTL3/elf3h/↑BRD4
METTL3 (Lee et al. 2019)	mHSCs	↓	↓			↑METTL3/↑MYC
METTL3 (Wu et al. 2020)	CLL		↓	↑		↓METTL3/↓SF3A1, SF3A2, SF3B1, U2AF1/↓ mTORC1
METTL3 (Cheng et al. 2020)	DLBCL		↓			↓METTL3/↓PEDF/↓Wnt pathway
METTL3/14 (Yao et al. 2018)	HSCs		↓			↓Mettl3/14/↓Nr4a2, p21, Bmi-1, and Prdm16
METTL3/14 (Wang et al. 2014a)	mESCs					↓METTL3/14/↓HuR/↓IGFBP3
METTL5 (Xing et al. 2020)	T-ALL				↑ leukemia-initiating cells (LICs)	↓METTL5/↓FBXW7/**↑**c-Myc
METTL16 (Pendleton et al. 2017)	MLL				↓MML progression	↓METTL16/↓MAT2A/↑DOT1L and PRMT5
WTAP (Kuai et al. 2018)	large B cell lymphoma		↓	↑		↓Hsp90/↓WTAP/↓BCL6
RBM15 (Raffel et al. 2007)	mB-cells	↓	↑			n/a
RBM15 (Patil et al. 2016)	mESCs				↓ Xist RNA	↓RBM15/↓Xist RNA/↓c-myc
m6A eraser						
ALKBH5 (Wang et al. 2020b)	LSCs				↓ leukemogenesis	↓KDM4C/↓MYC/PolII/↓ALKBH5/↓AXL
ALKBH5 (Liu et al. 2021a)	mice BM cells				↓ ILC3 homeostasis	↓circZbtb20/↓ALKBH5/↓Nr4a1/↓Notch2 signaling
ALKBH5 (Gong et al. 2021)	T-ALL		↓	↑	↓ cell invasion	↓ALKBH5/↑USP1/↑AuroraB
FTO (ALKBH9) (Zhang et al. 2020)	CLL		↓	↑		↓FTO/↓YTHDF2/↓Bcl-2/↑cleaved-PARP and BAX
m6A reader						
YTHDF2 (Wang et al. 2018a)	HSCs		↑			↓YTHDF2/↑Wnt target genes
YTHDF2 (Li et al. 2018)	mHSCs		↑			↓YTHDF2/↑Gata2, Etv6, Runx1, Stat5, and Tal1
YTHDF2 (Mapperley et al. 2021)	mHSCs	↑ myeloid bias			↑ proinflammatory pathway	↓YTHDF2/↑pStat1, pSTAT3, Il6ra, and Gadd45g
YTHDF2 (Turner et al. 2021)	B cells	↓				↓YTHDF2/↑Bach2, Pax5, Irf8 and Spi1
YTHDF2 (Zheng et al. 2020)	OP9 cells stromal cells		↓			↓YTHDF2/↑Zfp87, Sertad3, and Trib1
YTHDF2 (Dixit et al. 2021)	DLBCL cells		↓			↓YTHDF2/↓ACER2-ceramide metabolic axis
IGF2BP1 (Elcheva et al. 2020)	leukemia cells				↓ Leukemogenesis	↓IGF2BP1/↓HOXB4, MYB, and ALDH1A1
IGF2BP1 (Stoskus et al. 2016)	t(12;21)(p13;q22)-positive ALL cells (REH)				↓ ALL progression	↓IGF2BP1/↓ETV6-RUNX1/↓RAC1/↓STAT3
IGF2BP1 (Sharma et al. 2021)	ETV6-RUNX1 positive B-ALL cells		↓			↓IGF2BP1/↑TNF alpha-NFκB and K-Ras pathways
IGF2BP3 (Palanichamy et al. 2016a)	B-ALL		↓	↑		↑IGF2BP3/↑Myc and Cdk6

In leukemia, m6A demonstrate ambivalent pro-leukemia or anti-leukemia effects, depending on the m6A modified RNA substrate and downstream signaling pathways. m6A promote leukemic genes such as SF3A1/2, HOXB4, ALDH1A1, LIN28B, MYC, DOT1L, and HK2, whereby contribute to the TNFα/NFκB, PEDF/Wnt/β-catenin and dACER2-ceramide metabolic pathways in favor of an oncogenic phenotypes. On the contrary, m6A on lncRNA *NBAT1* blocks the interaction between an oncogene and its regulator, m6A on rRNA activates the ribosome and promotes tumor suppressor gene transcription, and m6A on mtRNA can inhibit leukemia cell growth through the RMRP-YBX1-TGF-βR1-SMAD axis. Each m6A regulatory axis can be leveraged as a druggable fulcrum in hematological diseases.

Future research directions lie within deciphering the interactions between the m6A machinery and lineage-specific regulations, developing small molecule m6A inhibitors to shift immune cell fate decisions, studying post-translational modifications (PTMs) of m6A writers, erasers, and readers (e.g., sumoylation of m6A readers, which decreases their stability and promotes solid cancer progression), and exploring m6A biology effects on non-coding RNAs.

## Data Availability

No datasets were generated or analysed during the current study.

## References

[CR1] Abla H, et al. The multifaceted contribution of α-ketoglutarate to tumor progression: an opportunity to exploit? Semin Cell Dev Biol. 2020;98:26–33. 31175937 10.1016/j.semcdb.2019.05.031

[CR2] Aguilo F, et al. Coordination of m(6)A mRNA methylation and gene transcription by ZFP217 regulates pluripotency and reprogramming. Cell Stem Cell. 2015;17(6):689–704. 26526723 10.1016/j.stem.2015.09.005 PMC4671830

[CR3] An Y, Duan H. The role of m6A RNA methylation in cancer metabolism. Mol Cancer. 2022;21(1):14. 35022030 10.1186/s12943-022-01500-4 PMC8753874

[CR4] Avner P, Heard E. X-chromosome inactivation: counting, choice and initiation. Nat Rev Genet. 2001;2(1):59–67. 11253071 10.1038/35047580

[CR5] Batista PJ, et al. m(6)A RNA modification controls cell fate transition in mammalian embryonic stem cells. Cell Stem Cell. 2014;15(6):707–19. 25456834 10.1016/j.stem.2014.09.019 PMC4278749

[CR6] Bell JL, et al. Insulin-like growth factor 2 mRNA-binding proteins (IGF2BPs): post-transcriptional drivers of cancer progression? Cell Mol Life Sci. 2013;70(15):2657–75. 23069990 10.1007/s00018-012-1186-z PMC3708292

[CR7] Boo SH, Kim YK. The emerging role of RNA modifications in the regulation of mRNA stability. Exp Mol Med. 2020;52(3):400–8. 32210357 10.1038/s12276-020-0407-z PMC7156397

[CR8] Cancro MP. Age-associated B cells. Annu Rev Immunol. 2020;38:315–40. 31986068 10.1146/annurev-immunol-092419-031130

[CR9] Chen MJ, et al. Runx1 is required for the endothelial to haematopoietic cell transition but not thereafter. Nature. 2009;457(7231):887–91. 19129762 10.1038/nature07619 PMC2744041

[CR10] Chen K, et al. High-resolution N(6) -methyladenosine (m(6) A) map using photo-crosslinking-assisted m(6) A sequencing. Angew Chem Int Ed Engl. 2015;54(5):1587–90. 25491922 10.1002/anie.201410647 PMC4396828

[CR11] Cheng Y, et al. m(6)A RNA methylation maintains hematopoietic stem cell identity and symmetric commitment. Cell Rep. 2019;28(7):1703-1716.e6. 31412241 10.1016/j.celrep.2019.07.032 PMC6818972

[CR12] Cheng Y, et al. The m6A methyltransferase METTL3 is functionally implicated in DLBCL development by regulating m6A modification in PEDF. Front Genet. 2020;11:955. 33061938 10.3389/fgene.2020.00955 PMC7481464

[CR13] Chitrakar A, et al. Introns encode dsRNAs undetected by RIG-I/MDA5/interferons and sensed via RNase L. Proc Natl Acad Sci U S A. 2021. 10.1073/pnas.2102134118. 10.1073/pnas.2102134118 PMC8609619 34772806

[CR14] Choe J, et al. mRNA circularization by METTL3-eIF3h enhances translation and promotes oncogenesis. Nature. 2018;561(7724):556–60. 30232453 10.1038/s41586-018-0538-8 PMC6234840

[CR15] Commerford SL, Carsten AL, Cronkite EP. Histone turnover within nonproliferating cells. Proc Natl Acad Sci U S A. 1982;79(4):1163–5. 6951165 10.1073/pnas.79.4.1163 PMC345921

[CR16] Dawson MA, Kouzarides T, Huntly BJ. Targeting epigenetic readers in cancer. N Engl J Med. 2012;367(7):647–57. 22894577 10.1056/NEJMra1112635

[CR17] Delaunay S, Frye M. RNA modifications regulating cell fate in cancer. Nat Cell Biol. 2019;21(5):552–9. 31048770 10.1038/s41556-019-0319-0

[CR18] Dixit D, et al. The RNA m6A reader YTHDF2 maintains oncogene expression and is a targetable dependency in glioblastoma stem cells. Cancer Discov. 2021;11(2):480–99. 33023892 10.1158/2159-8290.CD-20-0331 PMC8110214

[CR19] Dominissini D, et al. Topology of the human and mouse m6A RNA methylomes revealed by m6A-seq. Nature. 2012;485(7397):201–6. 22575960 10.1038/nature11112

[CR20] Dreyfuss G, Kim VN, Kataoka N. Messenger-RNA-binding proteins and the messages they carry. Nat Rev Mol Cell Biol. 2002;3(3):195–205. 11994740 10.1038/nrm760

[CR21] Du H, et al. YTHDF2 destabilizes m6A-containing RNA through direct recruitment of the CCR4–NOT deadenylase complex. Nat Commun. 2016;7(1):12626. 27558897 10.1038/ncomms12626 PMC5007331

[CR22] Elcheva IA, et al. RNA-binding protein IGF2BP1 maintains leukemia stem cell properties by regulating HOXB4, MYB, and ALDH1A1. Leukemia. 2020;34(5):1354–63. 31768017 10.1038/s41375-019-0656-9 PMC7196026

[CR23] Evans JR, et al. Members of the poly (rC) binding protein family stimulate the activity of the c-myc internal ribosome entry segment in vitro and in vivo. Oncogene. 2003;22(39):8012–20. 12970749 10.1038/sj.onc.1206645

[CR24] Fedeles BI, et al. The AlkB family of Fe(II)/α-Ketoglutarate-dependent dioxygenases: repairing nucleic acid alkylation damage and beyond. J Biol Chem. 2015;290(34):20734–42. 26152727 10.1074/jbc.R115.656462 PMC4543635

[CR25] Fei DL, et al. Impaired hematopoiesis and leukemia development in mice with a conditional knock-in allele of a mutant splicing factor gene U2af1. Proc Natl Acad Sci U S A. 2018;115(44):E10437-e10446. 30322915 10.1073/pnas.1812669115 PMC6217397

[CR26] Feng C, et al. Crystal structures of the human RNA demethylase Alkbh5 reveal basis for substrate recognition. J Biol Chem. 2014;289(17):11571–83. 24616105 10.1074/jbc.M113.546168 PMC4002068

[CR27] Gallardo M, et al. Uncovering the role of RNA-binding protein hnRNP K in B-cell lymphomas. J Natl Cancer Inst. 2020;112(1):95–106. 31077320 10.1093/jnci/djz078 PMC7489062

[CR28] Garcia-Campos MA, et al. Deciphering the “m(6)A Code” via antibody-independent quantitative profiling. Cell. 2019;178(3):731-747.e16. 31257032 10.1016/j.cell.2019.06.013

[CR29] Ge R, et al. m(6)A-SAC-seq for quantitative whole transcriptome m(6)A profiling. Nat Protoc. 2023;18(2):626–57. 36434097 10.1038/s41596-022-00765-9 PMC9918705

[CR30] Gong H, et al. ALKBH5-mediated m6A-demethylation of USP1 regulated T-cell acute lymphoblastic leukemia cell glucocorticoid resistance by Aurora B. Mol Carcinog. 2021;60(9):644–57. 34169564 10.1002/mc.23330

[CR31] Grenov AC, et al. The germinal center reaction depends on RNA methylation and divergent functions of specific methyl readers. J Exp Med. 2021. 10.1084/jem.20210360. 10.1084/jem.20210360 PMC8374864 34402854

[CR32] Grozhik AV, et al. Mapping m(6)A at individual-nucleotide resolution using crosslinking and immunoprecipitation (miCLIP). Methods Mol Biol. 2017;1562:55–78. 28349454 10.1007/978-1-4939-6807-7_5 PMC5562447

[CR33] Gu G, et al. IGF2BP1: a novel IGH translocation partner in B acute lymphoblastic leukemia. Cancer Genet. 2014;207(7–8):332–4. 25195122 10.1016/j.cancergen.2014.07.002

[CR34] Gu Y, et al. DMDRMR-mediated regulation of m(6)A-modified CDK4 by m(6)A reader IGF2BP3 drives ccRCC progression. Cancer Res. 2021;81(4):923–34. 33293428 10.1158/0008-5472.CAN-20-1619

[CR35] Han H, et al. piRNA-30473 contributes to tumorigenesis and poor prognosis by regulating m6A RNA methylation in DLBCL. Blood. 2021;137(12):1603–14. 32967010 10.1182/blood.2019003764

[CR36] Hansen TH, Bouvier M. MHC class I antigen presentation: learning from viral evasion strategies. Nat Rev Immunol. 2009;9(7):503–13. 19498380 10.1038/nri2575

[CR37] Hartmann AM, et al. The interaction and colocalization of Sam68 with the splicing-associated factor YT521-B in nuclear dots is regulated by the Src family kinase p59(fyn). Mol Biol Cell. 1999;10(11):3909–26. 10564280 10.1091/mbc.10.11.3909 PMC25688

[CR38] Hendra C, et al. Detection of m6A from direct RNA sequencing using a multiple instance learning framework. Nat Methods. 2022;19(12):1590–8. 36357692 10.1038/s41592-022-01666-1 PMC9718678

[CR39] Hsu PJ, He C. High-resolution mapping of N (6)-methyladenosine using m(6)A crosslinking immunoprecipitation sequencing (m(6)A-CLIP-Seq). Methods Mol Biol. 2019;1870:69–79. 30539547 10.1007/978-1-4939-8808-2_5 PMC6530771

[CR40] Huang H, et al. Recognition of RNA N(6)-methyladenosine by IGF2BP proteins enhances mRNA stability and translation. Nat Cell Biol. 2018;20(3):285–95. 29476152 10.1038/s41556-018-0045-z PMC5826585

[CR41] Huang H, et al. Histone H3 trimethylation at lysine 36 guides m6A RNA modification co-transcriptionally. Nature. 2019a;567(7748):414–9. 30867593 10.1038/s41586-019-1016-7 PMC6438714

[CR42] Huang S, et al. Insulin-like growth factor 2 mRNA binding protein 2 promotes aerobic glycolysis and cell proliferation in pancreatic ductal adenocarcinoma via stabilizing GLUT1 mRNA. Acta Biochim Biophys Sin (Shanghai). 2019b;51(7):743–52. 31089713 10.1093/abbs/gmz048

[CR43] Huang Y, et al. Small-molecule targeting of oncogenic FTO demethylase in acute myeloid leukemia. Cancer Cell. 2019c;35(4):677-691.e10. 30991027 10.1016/j.ccell.2019.03.006 PMC6812656

[CR44] Huang H, et al. Mettl14-mediated m6A modification is essential for germinal center B cell response. J Immunol. 2022;208(8):1924–36. 35365563 10.4049/jimmunol.2101071

[CR45] Ichikawa M, et al. A role for RUNX1 in hematopoiesis and myeloid leukemia. Int J Hematol. 2013;97(6):726–34. 23613270 10.1007/s12185-013-1347-3

[CR46] Iwafuchi H, et al. Seminoma accompanying mature teratoma in an infantile mediastinal region: a rare presentation of infantile germ cell tumors. Pathol Int. 2016;66(9):540–2. 27450779 10.1111/pin.12436

[CR47] Jia G, et al. N6-Methyladenosine in nuclear RNA is a major substrate of the obesity-associated FTO. Nat Chem Biol. 2011;7(12):885–7. 22002720 10.1038/nchembio.687 PMC3218240

[CR48] Jones AN, et al. Structural effects of m6A modification of the Xist A-repeat AUCG tetraloop and its recognition by YTHDC1. Nucleic Acids Res. 2022;50(4):2350–62. 35166835 10.1093/nar/gkac080 PMC8887474

[CR49] Jonkhout N, et al. The RNA modification landscape in human disease. RNA. 2017;23(12):1754–69. 28855326 10.1261/rna.063503.117 PMC5688997

[CR50] Karnell JL, et al. Role of CD11c(+) T-bet(+) B cells in human health and disease. Cell Immunol. 2017;321:40–5. 28756897 10.1016/j.cellimm.2017.05.008

[CR51] Kasal DN, et al. A Gata3 enhancer necessary for ILC2 development and function. Proc Natl Acad Sci U S A. 2021;118(32):e2106311118. 34353913 10.1073/pnas.2106311118 PMC8364216

[CR52] King B, et al. The ubiquitin ligase FBXW7 modulates leukemia-initiating cell activity by regulating MYC stability. Cell. 2013;153(7):1552–66. 23791182 10.1016/j.cell.2013.05.041 PMC4146439

[CR53] Klein U, Dalla-Favera R. Germinal centres: role in B-cell physiology and malignancy. Nat Rev Immunol. 2008;8(1):22–33. 18097447 10.1038/nri2217

[CR54] Kuai Y, et al. Wilms’ tumor 1-associating protein plays an aggressive role in diffuse large B-cell lymphoma and forms a complex with BCL6 via Hsp90. Cell Commun Signal. 2018;16(1):50. 30143009 10.1186/s12964-018-0258-6 PMC6108153

[CR55] Kuksa PP, et al. In Silico Identification of RNA Modifications from High-Throughput Sequencing Data Using HAMR. Methods Mol Biol. 2017;1562:211–29. 28349463 10.1007/978-1-4939-6807-7_14 PMC7233376

[CR56] Lan T, et al. KIAA1429 contributes to liver cancer progression through N6-methyladenosine-dependent post-transcriptional modification of GATA3. Mol Cancer. 2019;18(1):186. 31856849 10.1186/s12943-019-1106-z PMC6921542

[CR57] Lancrin C, et al. GFI1 and GFI1B control the loss of endothelial identity of hemogenic endothelium during hematopoietic commitment. Blood. 2012;120(2):314–22. 22668850 10.1182/blood-2011-10-386094

[CR58] Lee H, et al. Stage-specific requirement for Mettl3-dependent m(6)A mRNA methylation during haematopoietic stem cell differentiation. Nat Cell Biol. 2019;21(6):700–9. 31061465 10.1038/s41556-019-0318-1 PMC6556891

[CR59] Lehmann C, Zeis M, Uharek L. Activation of natural killer cells with interleukin 2 (IL-2) and IL-12 increases perforin binding and subsequent lysis of tumour cells. Br J Haematol. 2001;114(3):660–5. 11552995 10.1046/j.1365-2141.2001.02995.x

[CR60] Lempereur A, et al. The TGFβ pathway is a key player for the endothelial-to-hematopoietic transition in the embryonic aorta. Dev Biol. 2018;434(2):292–303. 29253505 10.1016/j.ydbio.2017.12.006

[CR61] Lewis K, Yoshimoto M, Takebe T. Fetal liver hematopoiesis: from development to delivery. Stem Cell Res Ther. 2021;12(1):139. 33597015 10.1186/s13287-021-02189-w PMC7890853

[CR62] Li HB, et al. m(6)A mRNA methylation controls T cell homeostasis by targeting the IL-7/STAT5/SOCS pathways. Nature. 2017;548(7667):338–42. 28792938 10.1038/nature23450 PMC5729908

[CR63] Li Z, et al. Suppression of m6A reader Ythdf2 promotes hematopoietic stem cell expansion. Cell Res. 2018;28(9):904–17. 30065315 10.1038/s41422-018-0072-0 PMC6123498

[CR64] Li J, et al. lncNBAT1/APOBEC3A is a mediator of HBX-induced chemoresistance in diffuse large B cell lymphoma cells. Mol Ther Nucleic Acids. 2022;27:1064–77. 35228900 10.1016/j.omtn.2022.01.015 PMC8850662

[CR65] Liu N, Pan T. N6-methyladenosine-encoded epitranscriptomics. Nat Struct Mol Biol. 2016;23(2):98–102. 26840897 10.1038/nsmb.3162

[CR66] Liu N, et al. Probing N6-methyladenosine RNA modification status at single nucleotide resolution in mRNA and long noncoding RNA. RNA. 2013;19(12):1848–56. 24141618 10.1261/rna.041178.113 PMC3884656

[CR67] Liu J, et al. A METTL3–METTL14 complex mediates mammalian nuclear RNA N6-adenosine methylation. Nat Chem Biol. 2014;10(2):93–5. 24316715 10.1038/nchembio.1432 PMC3911877

[CR68] Liu N, et al. N6-methyladenosine-dependent RNA structural switches regulate RNA–protein interactions. Nature. 2015;518(7540):560–4. 25719671 10.1038/nature14234 PMC4355918

[CR69] Liu Y, et al. Wtapblock Cell Differentiation of Hematopoietic Stem and Progenitor Cells. Blood. 2020;136(Supplement 1):30–30.

[CR70] Liu B, et al. Circular RNA circZbtb20 maintains ILC3 homeostasis and function via Alkbh5-dependent m6A demethylation of Nr4a1 mRNA. Cell Mol Immunol. 2021a;18(6):1412–24. 33911218 10.1038/s41423-021-00680-1 PMC8166869

[CR71] Liu X, et al. Novel associations between METTL3 gene polymorphisms and pediatric acute lymphoblastic leukemia: a five-center case-control study. Front Oncol. 2021b;11:635251. 34568001 10.3389/fonc.2021.635251 PMC8459019

[CR72] Liu C, et al. Absolute quantification of single-base m(6)A methylation in the mammalian transcriptome using GLORI. Nat Biotechnol. 2023;41(3):355–66. 36302990 10.1038/s41587-022-01487-9

[CR73] Lizama CO, et al. Repression of arterial genes in hemogenic endothelium is sufficient for haematopoietic fate acquisition. Nat Commun. 2015;6:7739. 26204127 10.1038/ncomms8739 PMC4519987

[CR74] Loder F, et al. B cell development in the spleen takes place in discrete steps and is determined by the quality of B cell receptor-derived signals. J Exp Med. 1999;190(1):75–89. 10429672 10.1084/jem.190.1.75 PMC2195560

[CR75] Lu TX, et al. A new model of spontaneous colitis in mice induced by deletion of an RNA m(6)A methyltransferase component METTL14 in T cells. Cell Mol Gastroenterol Hepatol. 2020;10(4):747–61. 32634481 10.1016/j.jcmgh.2020.07.001 PMC7498954

[CR76] Luo A, et al. Genetic variants in METTL14 are associated with the risk of acute lymphoblastic leukemia in southern chinese children: a five-center case-control study. Cancer Manag Res. 2021;13:9189–200. 34934362 10.2147/CMAR.S335925 PMC8684373

[CR77] Lv J, et al. Endothelial-specific m6A modulates mouse hematopoietic stem and progenitor cell development via Notch signaling. Cell Res. 2018;28(2):249–52. 29148543 10.1038/cr.2017.143 PMC5799811

[CR78] Ma S, et al. Pigment epithelium-derived factor alleviates endothelial injury by inhibiting Wnt/β-catenin pathway. Lipids Health Dis. 2017;16(1):31. 28173817 10.1186/s12944-017-0407-8 PMC5297210

[CR79] Ma S, et al. The interplay between m6A RNA methylation and noncoding RNA in cancer. J Hematol Oncol. 2019;12(1):121. 31757221 10.1186/s13045-019-0805-7 PMC6874823

[CR80] Ma S, et al. The RNA m6A reader YTHDF2 controls NK cell antitumor and antiviral immunity. J Exp Med. 2021. 10.1084/jem.20210279 10.1084/jem.20210279 PMC8225680 34160549

[CR81] Manesia JK, et al. Highly proliferative primitive fetal liver hematopoietic stem cells are fueled by oxidative metabolic pathways. Stem Cell Res. 2015;15(3):715–21. 26599326 10.1016/j.scr.2015.11.001

[CR82] Mapperley C, et al. The mRNA m6A reader YTHDF2 suppresses proinflammatory pathways and sustains hematopoietic stem cell function. J Exp Med. 2021. 10.1084/jem.20200829. 10.1084/jem.20200829 PMC7653684 33156926

[CR83] Masoud R, et al. The complementary role of insulin-like growth factor II mRNA-binding protein 3 (IMP3) in diagnosis of Hodgkin’s lymphoma. Ann Diagn Pathol. 2019;42:64–8. 31319330 10.1016/j.anndiagpath.2019.06.006

[CR84] Meyer KD. DART-seq: an antibody-free method for global m6A detection. Nat Methods. 2019;16(12):1275–80. 31548708 10.1038/s41592-019-0570-0 PMC6884681

[CR85] Miranda Furtado CL, et al. Epidrugs: targeting epigenetic marks in cancer treatment. Epigenetics. 2019;14(12):1164–76. 31282279 10.1080/15592294.2019.1640546 PMC6791710

[CR86] Mjösberg J, Spits H. Human innate lymphoid cells. J Allergy Clin Immunol. 2016;138(5):1265–76. 27677386 10.1016/j.jaci.2016.09.009

[CR87] Molinie B, et al. m6A-LAIC-seq reveals the census and complexity of the m6A epitranscriptome. Nat Methods. 2016;13(8):692–8. 27376769 10.1038/nmeth.3898 PMC5704921

[CR88] Morales AA, et al. Distribution of Bim determines Mcl-1 dependence or codependence with Bcl-xL/Bcl-2 in Mcl-1-expressing myeloma cells. Blood. 2011;118(5):1329–39. 21659544 10.1182/blood-2011-01-327197 PMC3152498

[CR89] Morita H, Moro K, Koyasu S. Innate lymphoid cells in allergic and nonallergic inflammation. J Allergy Clin Immunol. 2016;138(5):1253–64. 27817797 10.1016/j.jaci.2016.09.011

[CR90] Nair L, et al. Mechanism of noncoding RNA-associated N(6)-methyladenosine recognition by an RNA processing complex during IgH DNA recombination. Mol Cell. 2021;81(19):3949-3964.e7. 34450044 10.1016/j.molcel.2021.07.037 PMC8571800

[CR91] Ottersbach K. Endothelial-to-haematopoietic transition: an update on the process of making blood. Biochem Soc Trans. 2019;47(2):591–601. 30902922 10.1042/BST20180320 PMC6490701

[CR92] Palanichamy JK, et al. RNA-binding protein IGF2BP3 targeting of oncogenic transcripts promotes hematopoietic progenitor proliferation. J Clin Invest. 2016;126(4):1495–511. 26974154 10.1172/JCI80046 PMC4811152

[CR93] Palanichamy JK, et al. Role of IGF2BP1 and target genes in ETV6-RUNX1 positive B-acute lymphoblastic leukemia. Blood. 2019;134:3818.

[CR94] Palmer DC, Restifo NP. Suppressors of cytokine signaling (SOCS) in T cell differentiation, maturation, and function. Trends Immunol. 2009;30(12):592–602. 19879803 10.1016/j.it.2009.09.009 PMC2787651

[CR95] Patil DP, et al. m6A RNA methylation promotes XIST-mediated transcriptional repression. Nature. 2016;537(7620):369–73. 27602518 10.1038/nature19342 PMC5509218

[CR96] Pendleton KE, et al. The U6 snRNA m(6)A methyltransferase METTL16 regulates SAM synthetase intron retention. Cell. 2017;169(5):824-835.e14. 28525753 10.1016/j.cell.2017.05.003 PMC5502809

[CR97] Peng F, et al. Oncogenic AURKA-enhanced N(6)-methyladenosine modification increases DROSHA mRNA stability to transactivate STC1 in breast cancer stem-like cells. Cell Res. 2021;31(3):345–61. 32859993 10.1038/s41422-020-00397-2 PMC8027457

[CR98] Pieper K, Grimbacher B, Eibel H. B-cell biology and development. J Allergy Clin Immunol. 2013;131(4):959–71. 23465663 10.1016/j.jaci.2013.01.046

[CR99] Possot C, et al. Notch signaling is necessary for adult, but not fetal, development of RORγt+ innate lymphoid cells. Nat Immunol. 2011;12(10):949–58. 21909092 10.1038/ni.2105

[CR100] Pyfrom S, et al. The dynamic epigenetic regulation of the inactive X chromosome in healthy human B cells is dysregulated in lupus patients. Proc Natl Acad Sci U S A. 2021;118(24):e2024624118. 34103397 10.1073/pnas.2024624118 PMC8214693

[CR101] Qing Y, et al. R-2-hydroxyglutarate attenuates aerobic glycolysis in leukemia by targeting the FTO/m(6)A/PFKP/LDHB axis. Mol Cell. 2021;81(5):922-939.e9. 33434505 10.1016/j.molcel.2020.12.026 PMC7935770

[CR102] Raffel GD, et al. Ott1(Rbm15) has pleiotropic roles in hematopoietic development. Proc Natl Acad Sci U S A. 2007;104(14):6001–6. 17376872 10.1073/pnas.0609041104 PMC1851606

[CR103] Raffel S, et al. BCAT1 restricts αKG levels in AML stem cells leading to IDHmut-like DNA hypermethylation. Nature. 2017;551(7680):384–8. 29144447 10.1038/nature24294

[CR104] Reavie L, et al. Regulation of c-Myc ubiquitination controls chronic myelogenous leukemia initiation and progression. Cancer Cell. 2013;23(3):362–75. 23518350 10.1016/j.ccr.2013.01.025 PMC3609428

[CR105] Ren X, et al. Single-cell imaging of m(6) a modified RNA using m(6) A-specific in situ hybridization mediated proximity ligation assay (m(6) AISH-PLA). Angew Chem Int Ed Engl. 2021;60(42):22646–51. 34291539 10.1002/anie.202109118

[CR106] Rozovski U, Keating M, Estrov Z. The significance of spliceosome mutations in chronic lymphocytic leukemia. Leuk Lymphoma. 2013;54(7):1364–6. 23270583 10.3109/10428194.2012.742528 PMC4176818

[CR107] Schmidlin H, Diehl SA, Blom B. New insights into the regulation of human B-cell differentiation. Trends Immunol. 2009;30(6):277–85. 19447676 10.1016/j.it.2009.03.008 PMC2792751

[CR108] Secker KA, et al. MAT2A as key regulator and therapeutic target in MLLr leukemogenesis. Cancers (Basel). 2020;12(5):1342. 32456310 10.3390/cancers12051342 PMC7281730

[CR109] Selberg S, et al. Rational design of novel anticancer small-molecule RNA m6A demethylase ALKBH5 inhibitors. ACS Omega. 2021;6(20):13310–20. 34056479 10.1021/acsomega.1c01289 PMC8158789

[CR110] Sharma G, et al. Synergism between IGF2BP1 and ETV6-RUNX1 in the pathogenesis of ETV6-RUNX1 Positive B-Acute lymphoblastic leukaemia. Blood. 2021;138:3483.

[CR111] Shen C, et al. RNA demethylase ALKBH5 selectively promotes tumorigenesis and cancer stem cell self-renewal in acute myeloid leukemia. Cell Stem Cell. 2020;27(1):64-80.e9. 32402250 10.1016/j.stem.2020.04.009 PMC7335338

[CR112] Shen W, et al. GLORI for absolute quantification of transcriptome-wide m(6)A at single-base resolution. Nat Protoc. 2024;19(4):1252–87. 38253658 10.1038/s41596-023-00937-1

[CR113] Shi H, et al. YTHDF3 facilitates translation and decay of N6-methyladenosine-modified RNA. Cell Res. 2017;27(3):315–28. 28106072 10.1038/cr.2017.15 PMC5339834

[CR114] Shi H, Wei J, He C. Where, when, and how: context-dependent functions of RNA methylation writers, readers, and erasers. Mol Cell. 2019;74(4):640–50. 31100245 10.1016/j.molcel.2019.04.025 PMC6527355

[CR115] Shu X, et al. A metabolic labeling method detects m6A transcriptome-wide at single base resolution. Nat Chem Biol. 2020;16(8):887–95. 32341503 10.1038/s41589-020-0526-9

[CR116] Song H, et al. METTL3-mediated m6A RNA methylation promotes the anti-tumour immunity of natural killer cells. Nat Commun. 2021;12(1):5522. 34535671 10.1038/s41467-021-25803-0 PMC8448775

[CR117] Song W, et al. ALKBH5-mediated N6-methyladenosine modification of TRERNA1 promotes DLBCL proliferation via p21 downregulation. Cell Death Discov. 2022;8(1):25. 35031597 10.1038/s41420-022-00819-7 PMC8760254

[CR118] Stoskus M, et al. Identification of characteristic IGF2BP expression patterns in distinct B-ALL entities. Blood Cells Mol Dis. 2011;46(4):321–6. 21414819 10.1016/j.bcmd.2011.02.005

[CR119] Stoskus M, Eidukaite A, Griskevicius L. Defining the significance of IGF2BP1 overexpression in t(12;21)(p13;q22)-positive leukemia REH cells. Leuk Res. 2016;47:16–21. 27239736 10.1016/j.leukres.2016.05.009

[CR120] Su R, et al. R-2HG exhibits anti-tumor activity by targeting FTO/m(6)A/MYC/CEBPA signaling. Cell. 2018;172(1–2):90-105.e23. 29249359 10.1016/j.cell.2017.11.031 PMC5766423

[CR121] Sun C, et al. The study of METTL3 and METTL14 expressions in childhood ETV6/RUNX1-positive acute lymphoblastic leukemia. Mol Genet Genomic Med. 2019;7(10):e00933. 31429529 10.1002/mgg3.933 PMC6785433

[CR122] Syrett CM, et al. Loss of Xist RNA from the inactive X during B cell development is restored in a dynamic YY1-dependent two-step process in activated B cells. PLoS Genet. 2017;13(10):e1007050. 28991910 10.1371/journal.pgen.1007050 PMC5648283

[CR123] Syrett CM, et al. Altered X-chromosome inactivation in T cells may promote sex-biased autoimmune diseases. JCI Insight. 2019;4(7):e126751. 30944248 10.1172/jci.insight.126751 PMC6483655

[CR124] Szczepanski MJ, et al. Interleukin-15 enhances natural killer cell cytotoxicity in patients with acute myeloid leukemia by upregulating the activating NK cell receptors. Cancer Immunol Immunother. 2010;59(1):73–9. 19526239 10.1007/s00262-009-0724-5 PMC3721322

[CR125] Thambyrajah R, et al. GFI1 proteins orchestrate the emergence of haematopoietic stem cells through recruitment of LSD1. Nat Cell Biol. 2016;18(1):21–32. 26619147 10.1038/ncb3276

[CR126] Trabanelli S, et al. Tumour-derived PGD2 and NKp30-B7H6 engagement drives an immunosuppressive ILC2-MDSC axis. Nat Commun. 2017;8(1):593. 28928446 10.1038/s41467-017-00678-2 PMC5605498

[CR127] Tran KA, Dillingham CM, Sridharan R. The role of α-ketoglutarate-dependent proteins in pluripotency acquisition and maintenance. J Biol Chem. 2019;294(14):5408–19. 30181211 10.1074/jbc.TM118.000831 PMC6462505

[CR128] Tsujikawa K, et al. Expression and sub-cellular localization of human ABH family molecules. J Cell Mol Med. 2007;11(5):1105–16. 17979886 10.1111/j.1582-4934.2007.00094.x PMC4401260

[CR129] Turner DJ, et al. The RNA m6A binding protein YTHDF2 promotes the B cell to plasma cell transition. bioRxiv. 2021. 10.1101/2021.07.21.453193.

[CR130] Uddin MB, Wang Z, Yang C. The m(6)A RNA methylation regulates oncogenic signaling pathways driving cell malignant transformation and carcinogenesis. Mol Cancer. 2021;20(1):61. 33814008 10.1186/s12943-021-01356-0 PMC8019509

[CR131] Uenishi GI, et al. NOTCH signaling specifies arterial-type definitive hemogenic endothelium from human pluripotent stem cells. Nat Commun. 2018;9(1):1828. 29739946 10.1038/s41467-018-04134-7 PMC5940870

[CR132] Wan W, et al. METTL3/IGF2BP3 axis inhibits tumor immune surveillance by upregulating N(6)-methyladenosine modification of PD-L1 mRNA in breast cancer. Mol Cancer. 2022;21(1):60. 35197058 10.1186/s12943-021-01447-y PMC8864846

[CR133] Wang Y, et al. N6-methyladenosine modification destabilizes developmental regulators in embryonic stem cells. Nat Cell Biol. 2014a;16(2):191–8. 24394384 10.1038/ncb2902 PMC4640932

[CR134] Wang X, et al. N6-methyladenosine-dependent regulation of messenger RNA stability. Nature. 2014b;505(7481):117–20. 24284625 10.1038/nature12730 PMC3877715

[CR135] Wang X, et al. N(6)-methyladenosine modulates messenger RNA translation efficiency. Cell. 2015;161(6):1388–99. 26046440 10.1016/j.cell.2015.05.014 PMC4825696

[CR136] Wang H, et al. Loss of YTHDF2-mediated m(6)A-dependent mRNA clearance facilitates hematopoietic stem cell regeneration. Cell Res. 2018a;28(10):1035–8. 30150673 10.1038/s41422-018-0082-y PMC6170435

[CR137] Wang Y, et al. N6-methyladenosine RNA modification regulates embryonic neural stem cell self-renewal through histone modifications. Nat Neurosci. 2018b;21(2):195–206. 29335608 10.1038/s41593-017-0057-1 PMC6317335

[CR138] Wang Y, et al. Antibody-free enzyme-assisted chemical approach for detection of N6-methyladenosine. Nat Chem Biol. 2020a;16(8):896–903. 32341502 10.1038/s41589-020-0525-x

[CR139] Wang J, et al. Leukemogenic chromatin alterations promote AML leukemia stem cells via a KDM4C-ALKBH5-AXL signaling axis. Cell Stem Cell. 2020b;27(1):81-97.e8. 32402251 10.1016/j.stem.2020.04.001

[CR140] Wang X, et al. N(6)-methyladenosine modification of MALAT1 promotes metastasis via reshaping nuclear speckles. Dev Cell. 2021;56(5):702-715.e8. 33609462 10.1016/j.devcel.2021.01.015

[CR141] Wang Y, et al. Advanced on-site and in vitro signal amplification biosensors for biomolecule analysis. TrAC, Trends Anal Chem. 2022;149:116565.

[CR142] Ward PS, et al. The common feature of leukemia-associated IDH1 and IDH2 mutations is a neomorphic enzyme activity converting alpha-ketoglutarate to 2-hydroxyglutarate. Cancer Cell. 2010;17(3):225–34. 20171147 10.1016/j.ccr.2010.01.020 PMC2849316

[CR143] Wei CM, Gershowitz A, Moss B. Methylated nucleotides block 5’ terminus of HeLa cell messenger RNA. Cell. 1975;4(4):379–86. 164293 10.1016/0092-8674(75)90158-0

[CR144] Wei L, Chen H, Su R. M6APred-EL: a sequence-based predictor for identifying n6-methyladenosine sites using ensemble learning. Mol Ther Nucleic Acids. 2018;12:635–44. 30081234 10.1016/j.omtn.2018.07.004 PMC6082921

[CR145] Wei AH, et al. Venetoclax plus LDAC for newly diagnosed AML ineligible for intensive chemotherapy: a phase 3 randomized placebo-controlled trial. Blood. 2020;135(24):2137–45. 32219442 10.1182/blood.2020004856 PMC7290090

[CR146] Wei L, et al. Long noncoding RNA NBAT1 suppresses hepatocellular carcinoma progression via competitively associating with IGF2BP1 and decreasing c-Myc expression. Hum Cell. 2021;34(2):539–49. 33387362 10.1007/s13577-020-00464-1

[CR147] Wen J, et al. Zc3h13 regulates nuclear RNA m(6)A methylation and mouse embryonic stem cell self-renewal. Mol Cell. 2018a;69(6):1028-1038.e6. 29547716 10.1016/j.molcel.2018.02.015 PMC5858226

[CR149] Wen S, et al. Novel combination of histone methylation modulators with therapeutic synergy against acute myeloid leukemia in vitro and in vivo. Cancer Lett. 2018b;413:35–45. 29069576 10.1016/j.canlet.2017.10.015

[CR150] Woodruff MC, et al. Extrafollicular B cell responses correlate with neutralizing antibodies and morbidity in COVID-19. Nat Immunol. 2020;21(12):1506–16. 33028979 10.1038/s41590-020-00814-z PMC7739702

[CR151] Wu R, et al. m6A methylation controls pluripotency of porcine induced pluripotent stem cells by targeting SOCS3/JAK2/STAT3 pathway in a YTHDF1/YTHDF2-orchestrated manner. Cell Death Dis. 2019;10(3):171. 30787270 10.1038/s41419-019-1417-4 PMC6382841

[CR152] Wu Y, et al. Integrative transcriptome and quantitative proteome analyses identify METTL3 as a key regulator for aberrant RNA processing in chronic lymphocytic leukemia. Blood. 2020;136(Supplement 1):12–12.

[CR153] Xiao Y, et al. An elongation- and ligation-based qPCR amplification method for the radiolabeling-free detection of locus-specific N(6) -methyladenosine modification. Angew Chem Int Ed Engl. 2018;57(49):15995–6000. 30345651 10.1002/anie.201807942

[CR154] Xie Y, et al. Transcriptome-wide profiling of N (6)-methyladenosine via a selective chemical labeling method. Chem Sci. 2022;13(41):12149–57. 36349098 10.1039/d2sc03181g PMC9600483

[CR155] Xing M, et al. The 18S rRNA m(6) A methyltransferase METTL5 promotes mouse embryonic stem cell differentiation. EMBO Rep. 2020;21(10):e49863. 32783360 10.15252/embr.201949863 PMC7534618

[CR156] Xue T, et al. PADI2-catalyzed MEK1 citrullination activates ERK1/2 and promotes IGF2BP1-mediated SOX2 mRNA stability in endometrial cancer. Adv Sci (Weinh). 2021;8(6):2002831. 33747724 10.1002/advs.202002831 PMC7967072

[CR157] Yan H, et al. Roles and mechanisms of the m6A reader YTHDC1 in biological processes and diseases. Cell Death Discov. 2022;8(1):237. 35501308 10.1038/s41420-022-01040-2 PMC9061745

[CR158] Yang X, Wong MPM, Ng RK. Aberrant DNA methylation in acute myeloid leukemia and its clinical implications. Int J Mol Sci. 2019;20(18):4576. 31527484 10.3390/ijms20184576 PMC6770227

[CR159] Yang Z, et al. RNA N6-methyladenosine reader IGF2BP3 regulates cell cycle and angiogenesis in colon cancer. J Exp Clin Cancer Res. 2020a;39(1):203. 32993738 10.1186/s13046-020-01714-8 PMC7523351

[CR160] Yang X, et al. METTL14 suppresses proliferation and metastasis of colorectal cancer by down-regulating oncogenic long non-coding RNA XIST. Mol Cancer. 2020b;19(1):46. 32111213 10.1186/s12943-020-1146-4 PMC7047419

[CR161] Yao QJ, et al. Mettl3–Mettl14 methyltransferase complex regulates the quiescence of adult hematopoietic stem cells. Cell Res. 2018;28(9):952–4. 30006613 10.1038/s41422-018-0062-2 PMC6123394

[CR162] Yao Y, et al. METTL3-dependent m6A modification programs T follicular helper cell differentiation. Nat Commun. 2021;12(1):1333. 33637761 10.1038/s41467-021-21594-6 PMC7910450

[CR163] Yeh C-H, Bellon M, Nicot C. FBXW7: a critical tumor suppressor of human cancers. Mol Cancer. 2018;17(1):115. 30086763 10.1186/s12943-018-0857-2 PMC6081812

[CR164] Yu B, et al. B cell-specific XIST complex enforces X-inactivation and restrains atypical B cells. Cell. 2021;184(7):1790-1803.e17. 33735607 10.1016/j.cell.2021.02.015 PMC9196326

[CR165] Zhang C, et al. Inhibition of endothelial ERK signalling by Smad1/5 is essential for haematopoietic stem cell emergence. Nat Commun. 2014;5:3431. 24614941 10.1038/ncomms4431

[CR166] Zhang C, et al. m6A modulates haematopoietic stem and progenitor cell specification. Nature. 2017a;549(7671):273–6. 28869969 10.1038/nature23883

[CR167] Zhang Z, et al. SF3B1 mutation is a prognostic factor in chronic lymphocytic leukemia: a meta-analysis. Oncotarget. 2017b;8(41):69916–23. 29050251 10.18632/oncotarget.19455 PMC5642526

[CR168] Zhang Z, et al. Single-base mapping of m(6)A by an antibody-independent method. Sci Adv. 2019;5(7):eaax0250. 31281898 10.1126/sciadv.aax0250 PMC6609220

[CR169] Zhang Y, et al. Targeting inhibition of N6-methyladenosine demethylase Fto displays potent anti-tumor activities in chronic lymphocytic leukemia. Blood. 2020;136:35–6.

[CR170] Zhang X, et al. YTHDF3 modulates hematopoietic stem cells by recognizing RNA m(6)A modification on Ccnd1. Haematologica. 2022;107(10):2381–94. 35112553 10.3324/haematol.2021.279739 PMC9521252

[CR171] Zheng Z, et al. Control of early B cell development by the RNA N(6)-methyladenosine methylation. Cell Rep. 2020;31(13):107819. 32610122 10.1016/j.celrep.2020.107819 PMC7371152

[CR172] Zhou J, et al. Inhibition of LIN28B impairs leukemia cell growth and metabolism in acute myeloid leukemia. J Hematol Oncol. 2017;10(1):138. 28693523 10.1186/s13045-017-0507-y PMC5504806

[CR173] Zhu Y, et al. The E3 ligase VHL promotes follicular helper T cell differentiation via glycolytic-epigenetic control. J Exp Med. 2019;216(7):1664–81. 31123085 10.1084/jem.20190337 PMC6605754

[CR174] Zhu P, et al. A novel hypoxic long noncoding RNA KB-1980E6.3 maintains breast cancer stem cell stemness via interacting with IGF2BP1 to facilitate c-Myc mRNA stability. Oncogene. 2021;40(9):1609–27. 33469161 10.1038/s41388-020-01638-9 PMC7932928

